# Supplementary material for: A compact model of Escherichia coli core and biosynthetic metabolism
Source: PLoS Comput Biol. 2025 Oct 13;21(10):e1013564. doi: 10.1371/journal.pcbi.1013564 (PMC12533975; doi:10.1371/journal.pcbi.1013564)
Supplement: S1 Text — (PDF) [file pcbi.1013564.s001.pdf]

A compact model of *Escherichia coli* core and biosynthetic metabolism
Supplementary Material

Contents

A Changes to reactions from the model *i*ML1515 **2**

B Computation of an equivalent biomass reaction **2**

C Differences in acetate production between *i*CH360 and *i*ML1515 **3**

D Computation of attributes using the knowledge graph **3**

D.1 Computation of molecular masses for all protein nodes . . . 3

D.2 Computation of gene-protein-reaction rules . . . 3

E Estimation of enzyme complex abundances **4**

F Adjustment of turnover numbers based on proteomics measurements across conditions **4**

F.1 Preliminaries . . . 5

F.2 Overview of the fitting procedure . . . 5

F.3 Obtaining a set of reference flux distributions . . . 5

F.4 Estimating typical enzyme efficiencies for the reference set of flux distributions . . . 6

F.5 Conversion to apparent turnover numbers . . . 8

F.6 Potential extensions . . . 8

Figures **9**

Tables **31**

References **36**

A Changes to reactions from the model *i*ML1515

After assembling the model as a subset of reactions from the genome-scale model *i*ML1515, a few minor corrections were applied to some of the reactions based on evidence gathered from the literature. Note, however, that these changes did not result from an exhaustive process of review of the parent model. The corrections include:

- In *i*CH360, the membrane-bound transhydrogenase reaction (THD2pp) only translocates 1 proton across the periplasmic membrane, as opposed to the 2 protons translocated in the *i*ML1515 reaction [1]
- The gene-protein rule (GPR) of gene *glxK* (b0514) was reassigned to GLYCK2, which produces 2-phosphoglycerate. Meanwhile, the reaction GLYCK from *i*ML1515 (which produces 3-phosphoglycerate) was removed [2, 3]
- The NADPH-dependent homoserine dehydrogenase reaction (HSDy) was made irreversible towards the homoserine production direction [4]. To avoid having a reaction that is restricted to negative flux values, the substrates and products of the reactions were flipped.
- The succinate transport reaction SUCCt1pp was made irreversible in the export direction, enforcing the use of the more thermodynamically favourable SUCCt2_2pp (which translocates two protons instead of one) for succinate import. Again, to avoid having a reaction that is restricted to negative flux values, substrate and products of SUCCt1pp were flipped.

B Computation of an equivalent biomass reaction

In constraint-based models of metabolism, the biomass reaction summarises the production of all molecules that are not explicitly described by the model. These are typically macromolecules such as proteins or polynucleotides. Which metabolites are drained from the model network towards these other parts of metabolism, and in what proportions, depends on what compounds are described by the model. Hence, starting from an existing model, taking only a subset of the reactions but leaving the biomass reaction unchanged would lead to inconsistencies.

To construct a biomass reaction for *i*CH360 that corresponds equivalently to the biomass reaction in *i*ML1515, the following method was used. First, we collected all the pathways required for the production of the components present in the *i*ML1515 biomass reaction, but not in our model (with the exception of a small number of compounds present with very small stoichiometry, which were excluded from this analysis for simplicity). These additional pathways (available in the repository supporting this manuscript) were manually curated based on available literature and database annotations to ensure they represent the most biologically relevant bioproduction route for each biomass component. By adding these pathways to *i*CH360, we obtained an extended model (*i*CH360$_{\mathrm{ext}}$), which was able to predict growth directly through the original biomass reaction.

With this extended model at hand, we can continue as follows. Let N and M denote the number of reactions and metabolites, respectively, in *i*CH360. Further, let $N_{\mathrm{ext}} > N$ denote the number of reactions in the extended model *i*CH360$_{\mathrm{ext}}$. A reference flux distribution $\mathbf{v}^*_{\mathrm{ref}} \in \mathbb{R}^{N_{\mathrm{ext}}}$ is computed on the extended model through FBA. We can express this flux vector as:

$$\mathbf{v}^*_{\mathrm{ref}} = \begin{pmatrix} \mathbf{v}^* \\ \mathbf{v}^*_+ \\ v^*_{\mathrm{BM}} \end{pmatrix} \tag{1}$$

where $\mathbf{v}^*$ is the subset of its fluxes corresponding to reactions present in *i*CH360, $\mathbf{v}^*_+$ is the subset of fluxes corresponding to the reactions added to create the extended model *i*CH360$_{\mathrm{ext}}$, and v^*_{BM} is the flux through the *i*ML1515 biomass reaction. If the fluxes $\mathbf{v}^*$ were to be imposed on *i*CH360 (which, at this stage, does not yet contain a biomass reaction), a number of metabolites would necessarily remain unbalanced, that is:

$$\mathbf{S}\ \mathbf{v}^* \neq \mathbf{0} \tag{2}$$

where $\mathbf{S} \in \mathbb{R}^{M \times N}$ denotes the stoichiometric matrix of *i*CH360. We now seek to define an "equivalent" biomass reaction that, when added to the *i*CH360 network, will drain or produce metabolites so as to balance equation (2). Let $\mathbf{r}_{\mathrm{eq}} \in \mathbb{R}^M$ denote the stoichiometry of such biomass reaction. We compute $\mathbf{r}_{\mathrm{eq}}$ as the additional column of $\mathbf{S}$ required to balance the system while achieving the same biomass flux of the reference distribution. That is, $\mathbf{r}_{\mathrm{eq}}$ satisfies:

$$\begin{pmatrix} \mathbf{S}\ \mathbf{r}_{\mathrm{eq}} \end{pmatrix} \begin{pmatrix} \mathbf{v}^* \\ v^*_{\mathrm{BM}} \end{pmatrix} = \mathbf{0} \tag{3}$$

which can be solved as:

$$\mathbf{r}_{\mathrm{eq}} = -\frac{1}{v^*_{\mathrm{BM}}} \mathbf{S} \mathbf{v}^* \tag{4}$$

The stoichiometry of this equivalent biomass reaction depends on the choice of reference flux distribution $\mathbf{v}^*_{\mathrm{ref}}$ and generally may differ for different growth conditions. This results from the fact that, depending on the condition,

the extended model may produce the same biomass component through different pathways, which would then be converted by our procedure into different equivalent costs of precursors in the sub-model. Nevertheless, the additional biosynthesis pathways we used to form the extended model do not allow for alternative routes to biomass; hence, we found the equivalent biomass reaction to be, in this case, unique across conditions.

C Differences in acetate production between *i*CH360 and *i*ML1515

As discussed in the main text (see Results: Range of metabolic conversions described by production envelopes), *i*CH360 predicts different acetate production yields than its genome-scale parent, *i*ML1515. In order to understand the cause of these differences, we investigated optimal acetate production routes in both models using FBA.

Under aerobic conditions, we found that the differences can be traced to different production abilities for acetyl-CoA, a precursor of acetate. In *i*CH360, acetyl-CoA is produced entirely from pyruvate oxidation via either the Pyruvate Dehydrogenase (PDH) or the Pyruvate-Formate-Lyase (PFL) reactions (Panel A in Fig G, left). On the other hand, *i*ML1515 can produce acetyl-CoA via a number of additional pathways not included in our model (Panel A in Fig G, right). These include: the degradation of threonine either directly to acetyl-CoA (THRD, GLYAT), or indirectly via acetaldehyde (THRA, THRD, ATHRDHr, THRA2), which is then converted to acetyl-CoA; the degradation of 2-deoxy-D-ribose 5-phosphate into acetaldehyde (DRPA), followed by conversion into acetyl-CoA; the degradation of Autoinducer-2 into acetyl-CoA (AI2K, PAI2I, PAI2T). Indeed, we found that, in the region of the production envelope where the two models diverge significantly, up to 50% of acetyl-CoA production in the genome-scale model is accounted for by these degradation pathways. This is in disagreement with the understanding of the existing literature that, under aerobic growth on glucose, most of the acetyl-CoA is produced from the oxidation of pyruvate [5, 6]. Confirming these findings, the simultaneous deletion of four reactions blocking these degradation pathways (DRPA, PAI2T, THRA, THRD) in *i*ML1515 brings aerobic acetate production to levels comparable to *i*CH360 (Panel B in Fig G).

In the anaerobic scenario, we found that the differences between the two models are exacerbated by the ability of the genome-scale model to produce more pyruvate (which in turn results in higher acetyl-CoA production) than *i*CH360. Investigating the cause of this difference, we found that, under anaerobic conditions, the *i*ML1515 solution involves the uptake of external CO_2 and its use as a sink for electrons produced in glycolysis (Panel C in Fig G), which is thermodynamically unrealistic under ambient CO_2 levels. Blocking CO_2 uptake reduces the maximal anaerobic pyruvate yield in the genome-scale model but does not fully close the gap with the production capabilities of our model (Panel D in Fig G), implying the existence of additional routes for anaerobic production of pyruvate that are not included in *i*CH360.

D Computation of attributes using the knowledge graph

Using the knowledge graph supporting the stoichiometric model of *i*CH360 (see the main text), a number of useful properties can be computed based on simple operations. Here, we outline how such an approach was used to i) compute the molecular mass of all enzyme complexes in the model, based on known masses for all polypeptides and ii) construct the Boolean rules (GPRs) linking all reactions and proteins in the graph to the model genes. For a description of the different types of nodes and edges mentioned in this section, see Table B and Table C.

D.1 Computation of molecular masses for all protein nodes

In order to compute the molecular masses of all protein nodes in the graph, the protein nodes corresponding to polypeptides were first annotated with their molecular masses, which are readily available in the EcoCyc database. Then the molecular masses of all other protein nodes are estimated recursively as follows. Let $\mathcal{I}$ denote the index set of all protein nodes and $\mathcal{C}(i) \in \mathcal{I}$ the index set of protein components of node i, i.e. all nodes connected to node i by a subunit composition relationship. The molecular mass of any protein node i, M_i, is computed as:

$$M_i = \begin{cases} \bar{M}_i & \text{if node } i \text{ is a polypeptide} \\ \sum_{k \in \mathcal{C}(i)} w_{ik} M_k & \text{otherwise} \end{cases} \tag{5}$$

where $\bar{M}_i$ denotes the (known) molecular mass of polypeptide node i and w_{ik} denotes the weight of the edge between i and k.

D.2 Computation of gene-protein-reaction rules

Boolean gene-protein-reaction (GPR) rules are a widely used tool defining a map between a genotype (set of active genes) and a phenotype (set of active reactions) in a metabolic model. Conventionally, this is achieved by assigning to each reaction a Boolean expression given in terms of genes in the model. In this section, we show how such expressions were computed for the reactions in *i*CH360 using the knowledge graph.

Starting from the leaves of the graph (genes), we construct, for each node, a Boolean expression describing its state (active/inactive, corresponding to a Boolean True/False) in terms of its children. The exact form of this Boolean expression depends on the type of the node (reaction, protein, or logical) and the type of edges connecting it to its neighbours (Fig V). Particularly:

- A polypeptide node is active if its associated gene is also active (Panel A in Fig V, left).
- A multimeric protein is active if all of its subunits (the child nodes connected to it by a "subunit composition" edge) are active (Panel A in Fig V, middle).
- A modified protein is active if its unmodified form (the child node connected to it by a "protein modification" edge) and its modification requirements (the child nodes connected to it by a "protein modification requirement" edge) are active (Panel A in Fig V, right).
- A logical AND (logical OR) node is active if all (any) of its child nodes are active (Panel B in Fig V).
- Finally, a reaction node is active if any of its catalysing isozymes (the child nodes connected to it via a "catalysis" edge) are active and, at the same time, all of its catalytic requirements (the child nodes connected to it via a "non-catalytic requirement" edge) are also active (Panel C in Fig V).

Using these definitions, the Boolean expression describing the state of a reaction can be written, ultimately, solely in terms of genes, enabling computation of conventional GPR rules and their incorporation in the standard metabolic model.

E Estimation of enzyme complex abundances

In order to estimate the abundances of all enzymes in the model from proteomics data, we use the model graph (see the main text) to construct a stoichiometric map between enzymes and polypeptides. This map takes the form of a matrix $\mathbf{E} \in \mathbb{R}^{n \times m}$, where n is the number of enzymes in the model and $m \geq n$ the number of polypeptides, such that $\mathbf{E}_{ij}$ denotes the stoichiometry of polypeptide j in enzyme i. Some polypeptides may be part of additional enzyme complexes that are not part of the model. Using the available annotation to the EcoCyc database, we identified 7 such polypeptides, mapping to 9 out-of-model complexes. If these out-of-model complexes were not taken into account, the abundance of model enzymes to which these polypeptides map would be overestimated. Hence, we constructed a matrix $\hat{\mathbf{E}}$ by augmenting $\mathbf{E}$ with additional rows corresponding to the identified out-of-model complexes.

With this mapping at hand, we assume that polypeptide abundances $\mathbf{p}$ are related to enzyme abundances $\mathbf{e}$ (including the required additional complexes not accounted for in the model) by

$$\mathbf{p} = \hat{\mathbf{E}}^{\top}\mathbf{e} \tag{6}$$

Hence, given a vector of experimental measurements of polypeptide abundances $\bar{\mathbf{p}}$, we estimate enzyme abundances by solving the non-negative least squares (NNLS) problem:

$$\begin{aligned} \min_{\mathbf{e}} \quad & ||\bar{\mathbf{p}} - \hat{\mathbf{E}}^{\top}\mathbf{e}||_2^2 \\ \text{s.t} \quad & \mathbf{e} \geq 0 \end{aligned} \tag{7}$$

F Adjustment of turnover numbers based on proteomics measurements across conditions

In this section, we outline the procedure used to adjust the turnover numbers in EC-iCH360 (see the main text) by fitting proteomic measurements across conditions. Briefly, our aim is to adjust the turnover numbers that parametrise the model so that enzyme allocation predictions obtained through the enzyme-constrained formulation of FBA (see Methods in the main text) match more closely experimental measurements of enzyme abundances. By simultaneously fitting experimental measurements across many growth conditions, we improve the robustness of the fitting procedure to experimental error and generate a condition-independent set of "typical" apparent turnover numbers that predict average trends of enzyme allocation across conditions. The output of our procedure is a set of typical enzyme efficiencies, one for each enzyme, estimated from proteomic data across conditions, as well as a set of condition-specific scaling factors that account for differences in total measured enzyme abundances between conditions. In section F.1 we rigorously define these parameters and state the main assumption underlying our heuristic. In sections F.2-F.5 we formulate a two-steps optimisation problem whose solution, upon a suitable reparameterization, yields data-fitted estimates of the desired parameters.

F.1 Preliminaries

Consider an enzyme i in a given metabolic state j. This enzyme catalyses a metabolic flux v_{ij} (in mmol/gDW/h) given by

$$v_{ij} = \kappa_{ij}\ c_{ij}^{\text{enz}} \tag{8}$$

where c_{ij}^{enz} is the enzyme concentration and κ_{ij} is the enzyme efficiency (also known as "apparent turnover number", k_{app}). The efficiency κ_{ij} is a positive rate (here in 1/h, but usually reported in 1/s). Since, by definition, it must be lower than the enzyme's turnover number $k_{\text{cat,i}}$, we write it as

$$\kappa_{ij} = \sigma_{ij}\ k_{\text{cat,i}} \tag{9}$$

where $\sigma_{ij} \in [0, 1]$ is a unitless "capacity usage" factor. In enzyme-constrained models, enzymes are often expressed by their mass abundance $e_i = M_i\ c_i^{\text{enz}}$ (in g/gDW) instead of enzyme concentrations, where M_i is the enzyme molecular mass (in g/mol), so we can write the flux as

$$v_{ij} = \frac{1}{a_{ij}}\ e_{ij} \tag{10}$$

where a_{ij} is the enzyme cost per catalysed flux, given by the molecular mass M_i divided by the enzyme efficiency κ_{ij}.

In principle, the capacity usage factors σ_{ij} (and therefore efficiencies) of enzymes may freely vary between growth conditions. However, as a heuristic, we here assume that they can be approximated as the product of two factors: an enzyme-specific term and a condition-specific one, that is:

$$\sigma_{ij} \approx \sigma_i \cdot \tau_j \tag{11}$$

Here, the enzyme-specific factor σ_i denotes the "typical" capacity usage factor of our enzyme in the range of conditions studied. The condition-specific term, τ_j, is a unitless scaling factor that simultaneously increases or decreases the efficiencies of all enzymes depending on the cell's growth conditions. By convention, we assume that the values of τ_j are centred around 1. Substituting (11) in (9), we obtain an equivalent expression (under our assumptions) for the apparent turnover number κ_{ij}:

$$\kappa_{ij} = \underbrace{\sigma_i\ k_{\text{cat,i}}}_{\kappa_i}\ \tau_j \tag{12}$$

where κ_i is a "typical apparent turnover number" that is condition-independent. Practically, the above assumption allows us to simplify the problem by reducing the number of parameters to be fitted from $I \cdot J$ to $I + J$, where I and J denote the total number of enzymes and conditions considered, respectively. Our heuristic assumption corresponds to the idea that "high-quality carbon sources" allow a cell to establish metabolic states in which enzyme efficiencies are generally high, allowing for large fluxes per enzyme abundance in all the reactions, and therefore for high cell growth rates. Probably, this heuristic would fail in some other cases, e.g. cases in which enzymes are specifically perturbed by enzyme inhibitors. But in fact, it turns out that our model, assuming a single "typical apparent turnover number" $k_{\text{app,i}}$ for each enzyme, yields very good enzyme allocation predictions. This is what would be expected if our heuristic assumption were correct, and it therefore supports our heuristic prediction.

We now describe how the estimates of (enzyme-specific) efficiencies κ_i and (condition specific) scaling factors τ_j for our model were obtained from model simulations and proteomics data.

F.2 Overview of the fitting procedure

Fitting the typical turnover parameters to proteomic data is, in general, not simple. Due to the linear programming formulation underlying the enzyme-constrained FBA problem, optimal flux distributions (and, by direct consequence, enzyme allocation predictions) are discontinuous over the turnover parameter space, making derivative-based searches through this space problematic from a numerical perspective. Similarly, the high dimensionality of the parameter space limits the applicability of gradient-free optimisation algorithms. Hence, we restrict ourselves to the (comparatively simpler) problem of adjusting turnover parameters for a fixed set of reference flux distributions across growth conditions, constraining our search to the portion of parameter space in which these reference flux vectors remain optimal for their respective conditions. This simplified fitting procedure thus consists of two steps. In the first part of the procedure, we use an initial parameter set to compute a set of reference flux distributions (one per growth condition) using enzyme-constrained FBA. In the second part, turnover parameters are fitted based on these reference flux distributions and experimental measurements of enzyme abundances.

F.3 Obtaining a set of reference flux distributions

Following the sMOMENT formulation of enzyme-constrained FBA [7], we consider a metabolic network with N reactions and M metabolites where all metabolic fluxes are positive (i.e. reversible reactions are split into forward and backward components) and at most one enzyme is associated with each reaction (see Methods in the main text for more information about how such unique mapping between reactions and catalysing enzymes was generated in our

case). Hence, we assume that the enzyme cost required to sustain flux v_i for a given growth condition j is given by $a_{ij}\ v_i$, where the cost per unit flux a_{ij} is given by:

$$a_{ij} = \begin{cases} \frac{M_i}{\kappa_{ij}} & \text{if reaction } i \text{ is enzymatic} \\ 0 & \text{otherwise} \end{cases} \tag{13}$$

Here, M_i is the molecular mass of the enzyme associated with the reaction, κ_{ij} is the condition-dependent enzyme efficiency, as defined in (12). Given an initial guess for the value of each κ_{ij}, we compute a reference flux distribution for the jth growth condition, $\mathbf{v}_j^*$ by fixing the biomass flux, v_{BM} to the experimentally measured rate and minimising the total enzyme cost:

$$\begin{aligned} \mathbf{v}_j^* = \arg\min_{\mathbf{v}} \quad & \mathbf{a}_j^\top \mathbf{v} \\ \text{s.t.} \quad & \mathbf{S}\ \mathbf{v} = \mathbf{0} & \text{(a)} \\ & \mathbf{B}_j\ \mathbf{v} \le \mathbf{b}_j & \text{(b)} \\ & v_{\mathrm{BM}} = \bar{v}_{\mathrm{BM},j} & \text{(c)} \\ & \mathbf{v} \ge \mathbf{0} \end{aligned} \tag{14}$$

Here, the objective $\mathbf{a}_j^\top \mathbf{v}$ is the total enzyme cost for the jth growth condition, $\bar{v}_{\mathrm{BM},j}$ is the experimentally measured growth rate for the condition, $\mathbf{S} \in \mathbb{R}^{\mathrm{M}\times\mathrm{N}}$ is the stoichiometric matrix of the network, and $\mathbf{B}_j \in \mathbb{R}^{\mathrm{P}\times\mathrm{N}}$ and $\mathbf{b}_j \in \mathbb{R}^{\mathrm{P}}$ are a matrix and a vector, respectively, encoding any desired upper bound (or positive lower bound) on the fluxes for the growth condition. Noting that constraint 14c can be equivalently cast as a double inequality, we rewrite the problem in the more general form:

$$\begin{aligned} \mathbf{v}_j^* = \arg\min_{\mathbf{v}} \quad & \mathbf{a}_j^\top \mathbf{v} \\ \text{s.t.} \quad & \mathbf{S}\ \mathbf{v} = \mathbf{0} & \text{(a)} \\ & \hat{\mathbf{B}}_j\ \mathbf{v} \le \hat{\mathbf{b}}_j & \text{(b)} \\ & \mathbf{v} \ge \mathbf{0} \end{aligned} \tag{15}$$

where the biomass flux is assumed to be the last component of the flux vector and the augmented matrices $\hat{\mathbf{B}}_j \in \mathbb{R}^{(\mathrm{P}+2)\times\mathrm{N}}$ and $\hat{\mathbf{b}}_j \in \mathbb{R}^{\mathrm{P}+2}$, defined as

$$\hat{\mathbf{B}}_j \equiv \begin{pmatrix} \mathbf{B_j} \\ [0, \cdots, \ \ 1] \\ [0, \cdots, -1] \end{pmatrix} \qquad \hat{\mathbf{b}}_j \equiv \begin{pmatrix} \mathbf{b}_j \\ \bar{v}_{\mathrm{BM},k} \\ -\bar{v}_{\mathrm{BM},k} \end{pmatrix} \tag{16}$$

were introduced.

In order to solve the linear program 14, we shall consider an initial guess for the turnover parameters and assume that, for each condition, all enzymes operate at the same saturation level, so that $\sigma_{ij} \equiv \bar{\sigma}_j$. Since the optimal flux distribution obtained as a solution of problem (15) is unchanged by the choice of $\bar{\sigma}_j$ (as this merely amounts to a scaling of the objective function), we can simply set $\bar{\sigma}_j = 1$ (that is, set $\kappa_{ij} = k_{\mathrm{cat,i}}$) for all conditions at this stage.

Solving (15) for each of the J growth conditions available in the experimental dataset, we obtain a set of optimal flux distributions $\mathcal{V}^* = \{\mathbf{v}_1^*, \cdots, \mathbf{v}_J^*\}$, which we will use as a reference in the next step.

F.4 Estimating typical enzyme efficiencies for the reference set of flux distributions

We now turn to fitting the relevant parameters against experimental measurements of enzyme abundances. For this purpose, we shall express the efficiency of each enzyme-condition pair as:

$$\kappa_{ij} = \theta_j\ k_i \tag{17}$$

where θ_j is a condition-specific scaling term that simultaneously scales all efficiencies for a given condition, while k_i is an "adjusted" turnover parameter that simultaneously accounts for inaccuracies in the original turnover parameter numbers as well as differences in typical saturation across enzymes (and, hence, it is not formally a turnover number). While the above parametrisation differs from the one in equation (12) – the terms have a different interpretation! – it will greatly simplify the notation of the fitting problem, as it allows us to easily distinguish between "global" adjustments (those affecting all enzymes in a condition), which we wish to pick freely, and "local" adjustments (affecting the efficiency of individual enzymes), which instead we wish to regularise. While this factorisation of saturation effects is merely a computational convenience and not necessarily biologically meaningful, we will retrieve the parameters in Eq. (12) from those in Eq. (17) upon a simple reparametrisation, as we detail in Section F.5

To formulate our fitting procedure, we will denote with $\mathbf{p}$ a vector of $\log_{10}$ adjusted turnover numbers (i.e. $p_i = \log_{10} k_i$) and with $\mathbf{s}$ a vector of condition-specific $\log_{10}$-scaling factors (i.e. $s_j = \log_{10}\theta_j$). Further, we denote with $\bar{\mathbf{p}}$

the vector of original log-turnover numbers (i.e. the one used to obtain the reference flux distributions). For the choice of reference flux distribution computed in the previous step, the abundance of the ith enzyme in condition j, e_{ij}, is then a function of $\mathbf{p}$ and $\mathbf{s}$:

$$e_{ij}(\mathbf{p}, \mathbf{s}) = \sum_{k} a_{kj}\ v^*_{kj} \tag{18}$$

where the summation index k runs across all reactions catalysed by enzyme i, and the flux cost of reaction k in condition j, a_{kj}, is computed as in (13). From the formulation of the linear program (15), there must exist a region $\mathcal{S}_j$ of log-turnover parameter space such that $\bar{\mathbf{p}} \in \mathcal{S}_j$ and that, for every $\mathbf{p} \in \mathcal{S}_j$, $\mathbf{v}^*_j \in \mathcal{V}^*$ is the optimal solution of problem (15) for its growth condition. Hence, in this step of the adjustment procedure, we aim to find a set of typical log efficiencies, $\mathbf{p}^*$ and log scaling factors, $\mathbf{s}^*$ by minimising the discrepancy between enzyme abundance predictions and measurements, constraining our search to this region of the parameter space $\mathcal{S} \equiv \bigcap_j \mathcal{S}_j$ where all reference flux distributions are optimal for their respective growth condition:

$$\begin{aligned} (\mathbf{p}^*, \mathbf{s}^*) = \underset{\mathbf{p},\ \mathbf{s}}{\arg\min}\quad & \frac{1}{N_\mathrm{e}} \sum_{i,j} \left[\ l(e_{ij}) - l(\bar{e}_{ij})\ \right]^2 + \frac{\rho}{N_\mathrm{p}} \sum_i \left(\ p_i - \bar{p}_i\ \right)^2 \\ \text{s.t.}\quad & \mathbf{u}_\mathrm{min} \le \mathbf{p} - \bar{\mathbf{p}} \le \mathbf{u}_\mathrm{max} \\ & \mathbf{p} \in \mathcal{S} \end{aligned} \tag{19}$$

where N_e is the number of enzyme-condition pairs, N_p is the number of turnover parameters, $\bar{e}_{ij}$ is the experimental measurement of enzyme i in condition j, $\mathbf{u}_\mathrm{min}$ and $\mathbf{u}_\mathrm{max}$ are bounds on the allowable adjustment, $\rho > 0$ is a scalar hyperparameter, and the function $l(\cdot)$ is defined as:

$$l(x) = \begin{cases} \log_{10}(x) & x > 0 \\ 0 & x = 0 \end{cases} \tag{20}$$

The objective function of the nonlinear program (19) is a combination of two terms. The first term penalises the mean squared deviation between measurements and predictions of log-enzyme abundance. Note that the above definition of $l(\cdot)$ implies that, for each condition, only enzymes with non-zero predicted abundance are included in this term. The second term is a regularisation expression (whose strength is controlled by the hyperparameter ρ) penalising the mean squared deviation between the adjusted turnover parameters and the original parameter set. The latter term mainly serves two purposes: first, it ensures that, whenever a turnover parameter is "free" in the problem (which can happen if its associated reaction fluxes are 0 across all conditions, or if no experimental measurements are available for its associated enzyme), it will be kept at its original value; secondly, it provides a mean to tune the strength of the adjustment procedure.

In order to define the region $\mathcal{S}$, we shall exploit the sufficient optimality conditions of LP (15). Introducing, for each growth condition, the vectors of dual variables, $\boldsymbol{\lambda}_j \in \mathbb{R}^M$ and $\boldsymbol{\mu}_j \in \mathbb{R}^{P+2}$, corresponding to constraints 15a and 15b, respectively, we note that problem (15) admits (for the jth growth condition) a dual problem in the form:

$$\begin{aligned} \max_{\boldsymbol{\lambda}_j,\ \boldsymbol{\mu}_j}\quad & -\hat{\mathbf{b}}_j^\top \boldsymbol{\mu}_j \\ \text{s.t.}\quad & \mathbf{S}^\top \boldsymbol{\lambda}_j\ + \hat{\mathbf{B}}_j^\top \boldsymbol{\mu}_j + \mathbf{a}_j \ge \mathbf{0} \qquad \text{(a)} \\ & \boldsymbol{\mu}_j \ge \mathbf{0} \end{aligned} \tag{21}$$

A well-known result in linear programming duality theory [8] states that a flux distribution is the optimal solution of the primal problem (15) if and only if the dual problem (21) is feasible (dual feasibility) and its optimal objective coincides with the primal optimal objective, that is $\mathbf{a}_j^\top \mathbf{v}_j^* = -\mathbf{b}_j^\top \boldsymbol{\mu}_j$ (strong duality). Taken together, dual feasibility and strong duality thus define the region of optimality in the space of turnover parameters of each reference flux distribution. Hence, we can integrate the above definition of $\mathcal{S}$ within problem (19) by introducing the two vectors of dual variables ($\boldsymbol{\lambda}_j$ and $\boldsymbol{\mu}_j$) for each condition as additional optimisation variables, and simultaneously enforcing the optimality for each reference distribution. By doing this, we obtain the final formulation of the nonlinear program for turnover number adjustment, which we solved to obtain the results shown in the main text:

$$\begin{aligned} (\mathbf{p}^*, \mathbf{s}^*) = \underset{\mathbf{p},\ \mathbf{s}}{\arg\min}\quad & \frac{1}{N_\mathrm{e}} \sum_{ij} \left[\ l(e_{ij}) - l(\bar{e}_{ij})\ \right]^2 + \frac{\rho}{N_\mathrm{p}} \sum_i \left(\ p_i - \bar{p}_i\ \right)^2 \\ \text{s.t.}\quad & \mathbf{u}_\mathrm{min} \le \mathbf{p} - \bar{\mathbf{p}} \le \mathbf{u}_\mathrm{max} \\ & \mathbf{S}^\top \boldsymbol{\lambda}_j\ + \hat{\mathbf{B}}_j^\top \boldsymbol{\mu}_j + \mathbf{a}_j \ge \mathbf{0} \qquad j = 1, \ldots, J \\ & \boldsymbol{\mu}_j \ge \mathbf{0} \qquad j = 1, \ldots, J \\ & \mathbf{a}_j^\top \mathbf{v}_j^* = -\hat{\mathbf{b}}_j^\top \boldsymbol{\mu}_j \qquad j = 1, \ldots, J \end{aligned} \tag{22}$$

F.5 Conversion to apparent turnover numbers

The above procedure produces (after conversion back to a linear scale) a set of adjusted turnover parameters, $\mathbf{k}^*$ and scalings, $\boldsymbol{\theta}^*$. In order to retrieve the typical enzyme efficiencies and condition-specific scaling factors introduced in 12, we simply parametrise the solution by factorising the scaling terms θ_j^* as

$$\theta_j^* \equiv \bar{\sigma}^* \ \tau_j^* \tag{23}$$

Here, $\bar{\sigma}^*$ is the geometric mean of the scalings across conditions, which we interpret as typical enzyme saturation level across conditions, while τ_j^* is a residual scaling factor fluctuating around 1 which is required to account for differences in total measured enzymes between conditions. The typical enzyme efficiencies (κ_i) introduced in 12 are then simply recovered by incorporating the $\bar{\sigma}^*$ constant into the fitted turnover parameters:

$$\kappa_i \equiv \bar{\sigma}^* \ k_i^* \tag{24}$$

F.6 Potential extensions

We conclude this section by noting that the procedure described above assumes that the original parameter set – the set of k_{cat} values used as proxies for apparent k_{cat} values in section F.3 – is sufficiently good to produce a realistic flux distribution to use as a reference for the adjustment step. If this is not the case, then a multi-start approach can be implemented, where multiple turnover parameter sets are first generated by perturbing the original parameter set, and each of them is used to generate a separate set of flux distributions. Each reference set is then provided as an input to problem (22), and the solution achieving the lowest objective is chosen in the end. The perturbed parameter set may be generated either randomly (for example, by introducing log-normal noise in the original turnover parameter vector) or systematically. The latter could be achieved, for example, by identifying reactions with zero-predicted flux but high measured abundance of the associated enzyme. By systematically increasing the corresponding turnover number, one can "encourage" these reactions to be included in the reference set, potentially leading to the exploration of more relevant reference distributions than what is achieved by random perturbations.

Note that in this work, we limited ourselves to the original parameter set and thus did not explore this potential heuristic.

Supplementary Figures

Biosynthesis of aminoacids

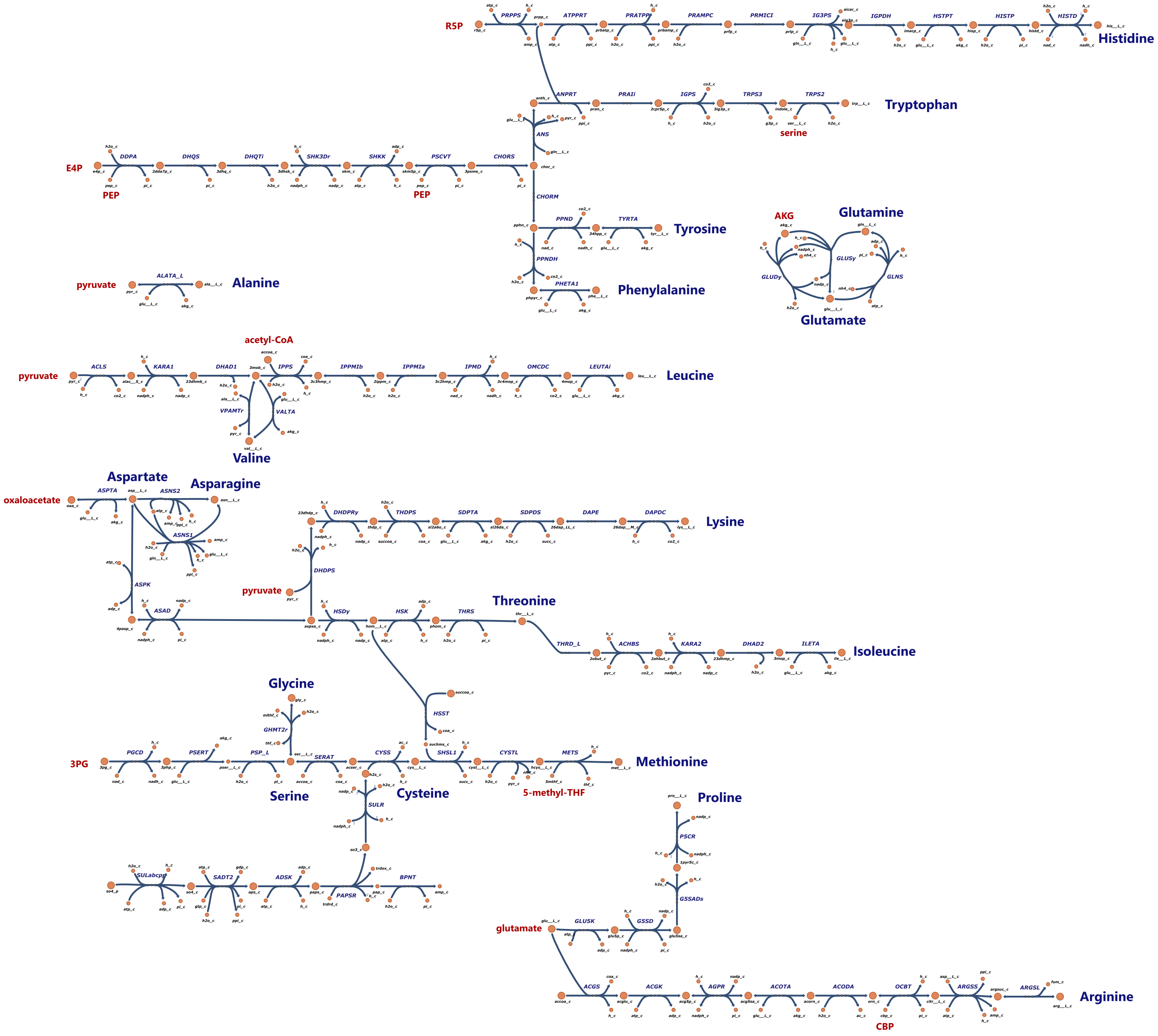

Fig A: Biosynthesis of amino acids (blue labels) from core metabolism precursors (red labels) in *i*CH360. The Escher maps (including Fig B to Fig D) are available along with the code. R5P: ribose-5-phosphate; E4P: erythrose 4-phosphate; PEP: phosphoenolpyruvate; 3PG: 3-phosphoglycerate; AKG: alpha-ketoglutarate; CBP: carbamoyl phosphate.

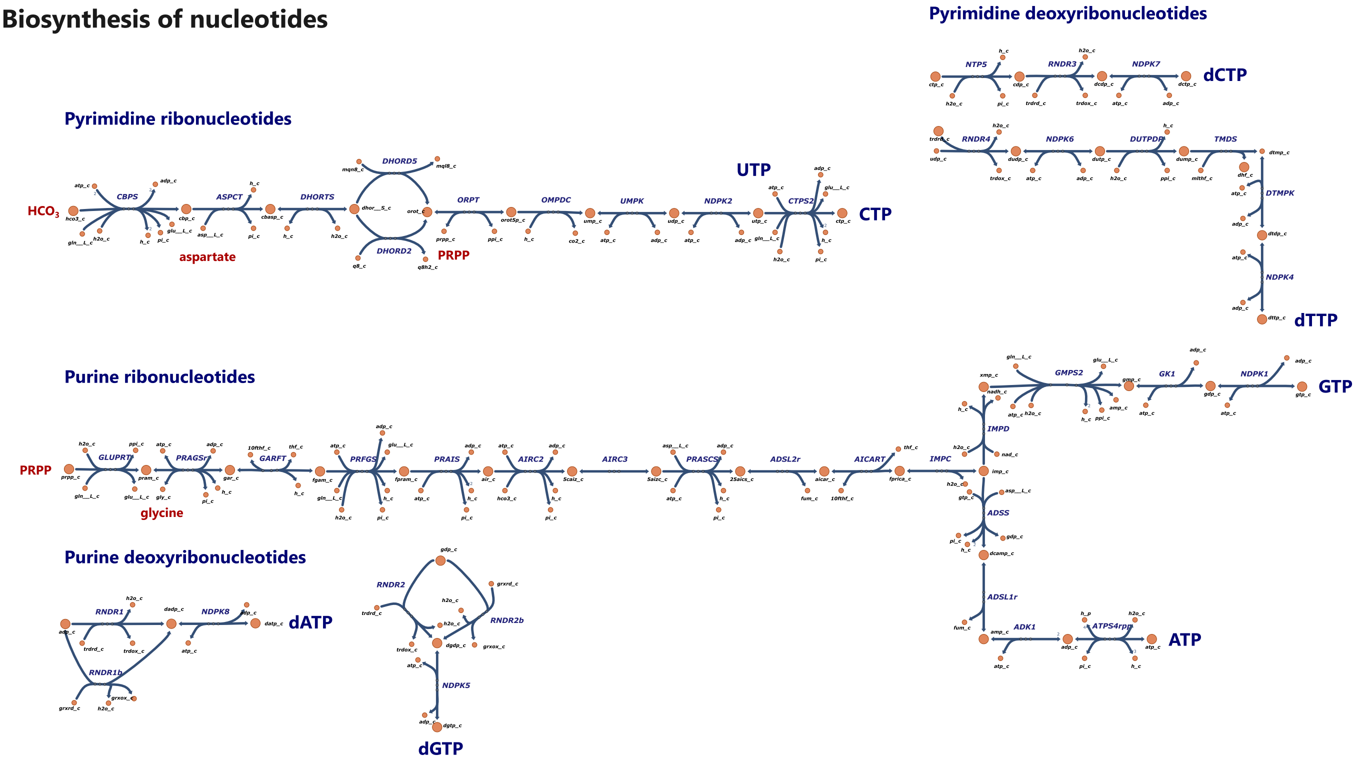

Fig B: Biosynthesis of pyrimidine and purine (deoxy)ribonucleotides (blue labels) from core and amino acid metabolism precursors (red labels) in *i*CH360. PRPP: 5-phosphoribosyl-1-pyrophosphate.

Biosynthesis of fatty acids

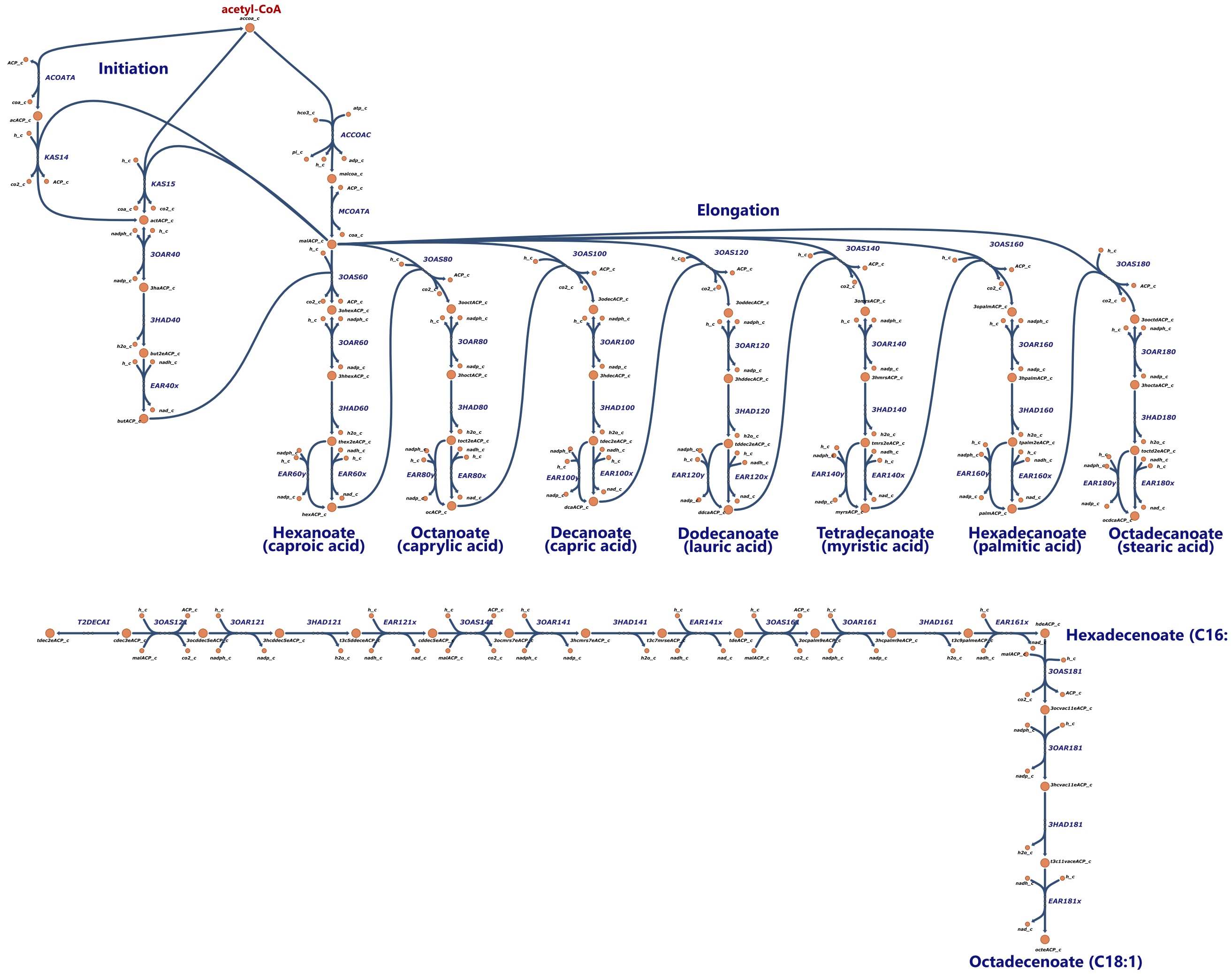

Fig C: Biosynthesis of saturated and unsaturated fatty acids from acetyl-CoA. The map for saturated fatty acids was taken from Escher [9].

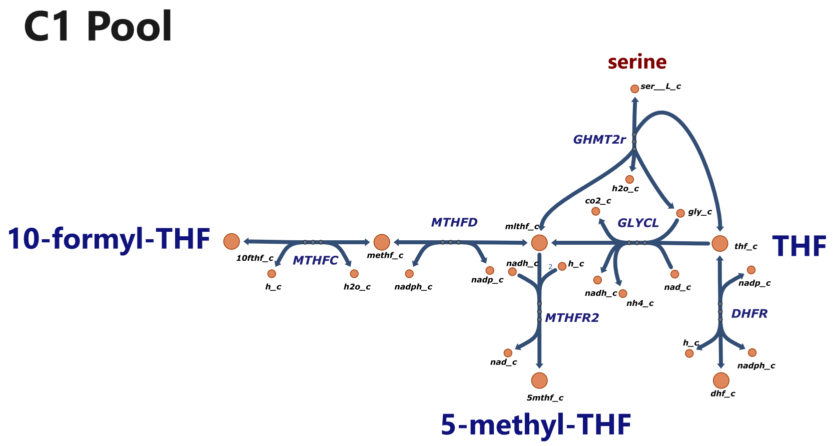

Fig D: Metabolism of one-carbon compounds in *i*CH360. THF: tetrahydrofolate.

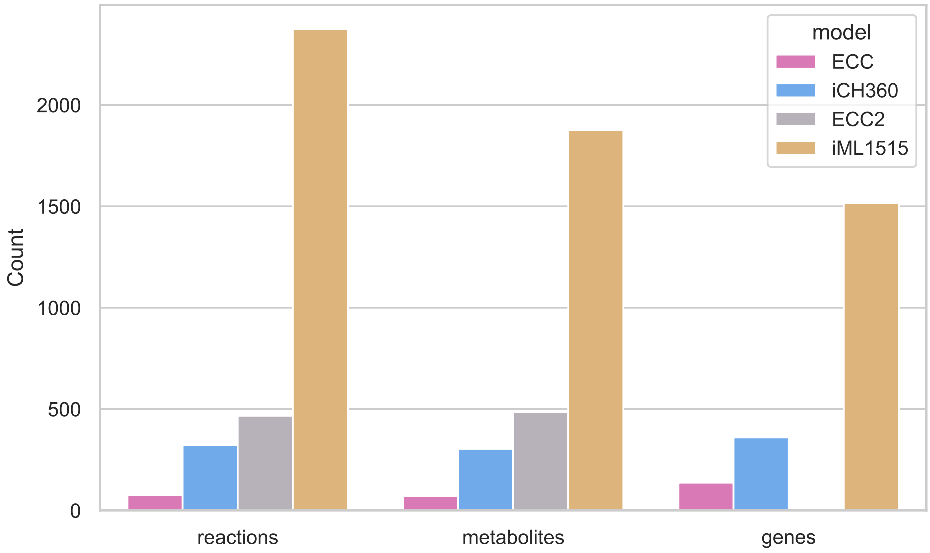

Fig E: Comparison of model sizes between ECC, ECC2, iCH360 and iML1515. To allow for a fair comparison, pseudo-reactions (e.g. exchange reactions) were excluded from the count. Note that gene annotations were not available in the SBML model of ECC2 accompanying its publication [10].

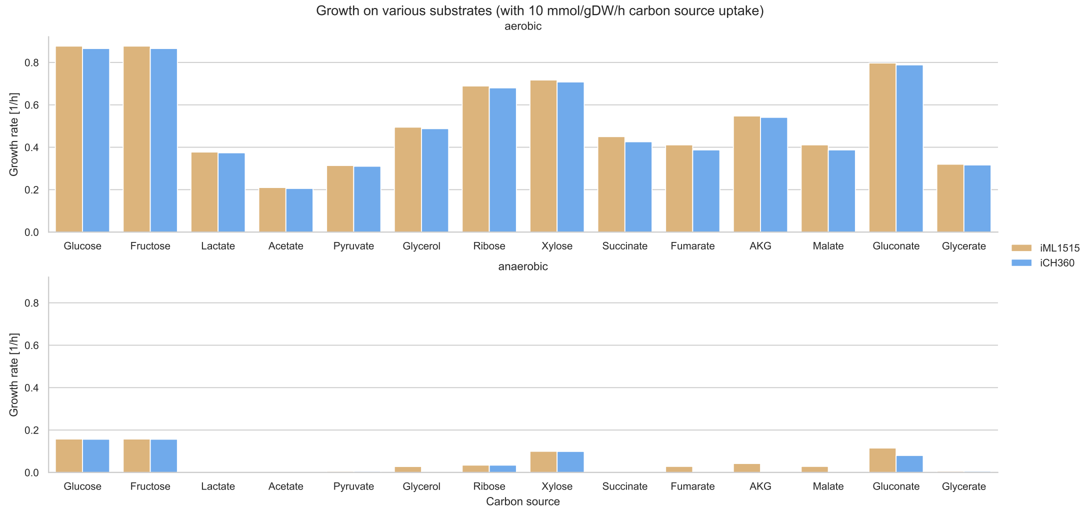

Fig F: Maximal biomass fluxes achieved by *i*CH360 and its parent model *i*ML1515, for aerobic and anaerobic growth across multiple carbon sources. In all cases, the substrate uptake flux was bounded to 10 mmol/gDW/h.

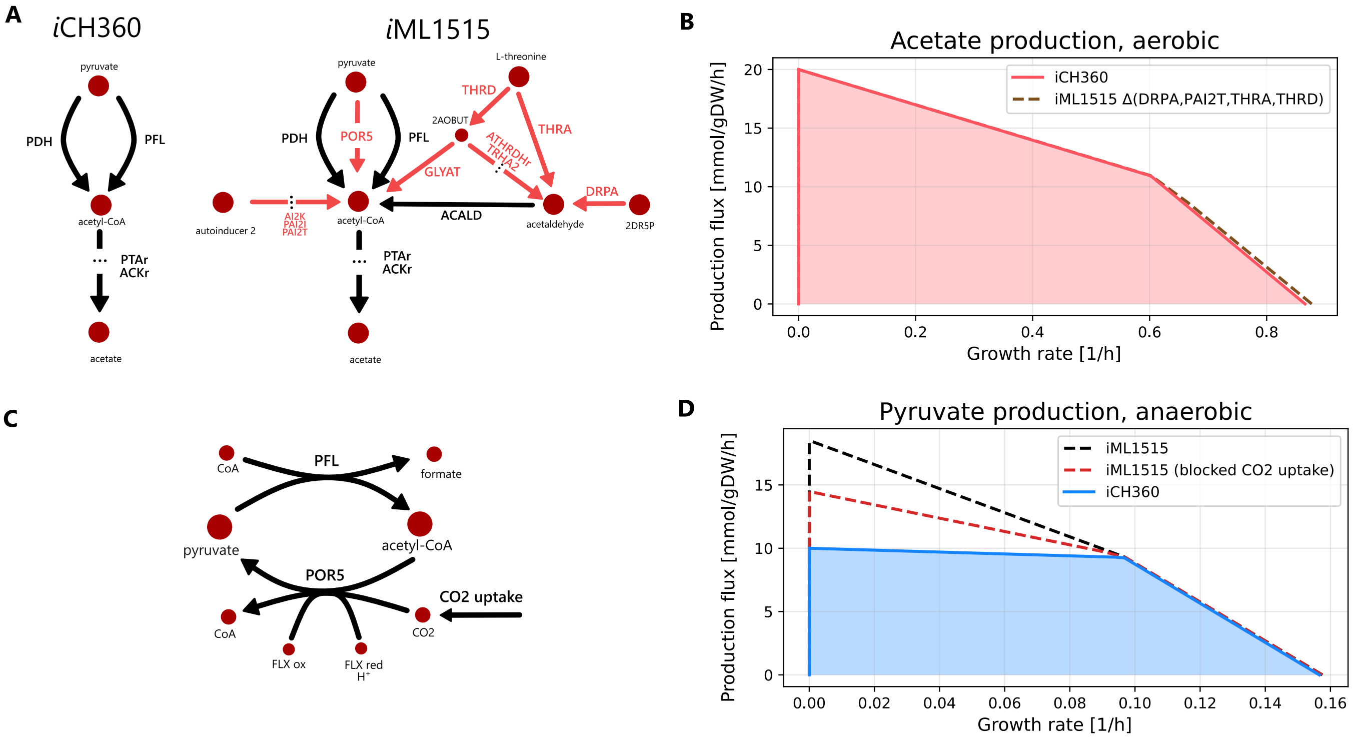

Fig G: Analysis of the differences in acetate production between *i*CH360 and its genome-scale parent, *i*ML1515. **A**: available metabolic routes for the production of acetate in both models. Left: *i*CH360 can only produce acetyl-CoA, precursor for acetate, via the oxidation of pyruvate by either pyruvate dehydrogenase (PDH) or pyruvate-formate-lyase (PFL). Note that the latter reaction is known not to be active under aerobic conditions, but we did not block it for the purpose of this analysis. *i*ML1515 can additionally produce acetate via additional pathways not present in *i*CH360 (in red). Note that only the main substrate and products for each reaction are shown for clarity. 2AOBUT: L-2-Amino-3-oxobutanoate; 2DR5P: 2-Deoxy-D-ribose 5-phosphate. **B**: Blocking these degradation routes by simultaneous knockout of four reactions (DRPA, PAI2T, THRA, THRD) results in the two models sharing a virtually identical production envelope under aerobic conditions. The production envelope shown here was computed for aerobic growth using glucose as a carbon source. **C**: Under anaerobic conditions, the differences between the two models are further exacerbated by the ability of the genome-scale network to achieve higher pyruvate production yield. The genome-scale model can produce higher amounts of pyruvate by uptaking external CO_2 and using it as an electron sink using the POR5 reaction. FLX ox/red: oxidised/reduced flavodoxin. **D**: Blocking CO_2 uptake reduces the differences in pyruvate production between the two models, but does not remove them completely, implying the existence of additional mechanisms used by *i*ML1515 to achieve higher pyruvate yields. The production envelopes shown here was computed for anaerobic growth using glucose as a carbon source.

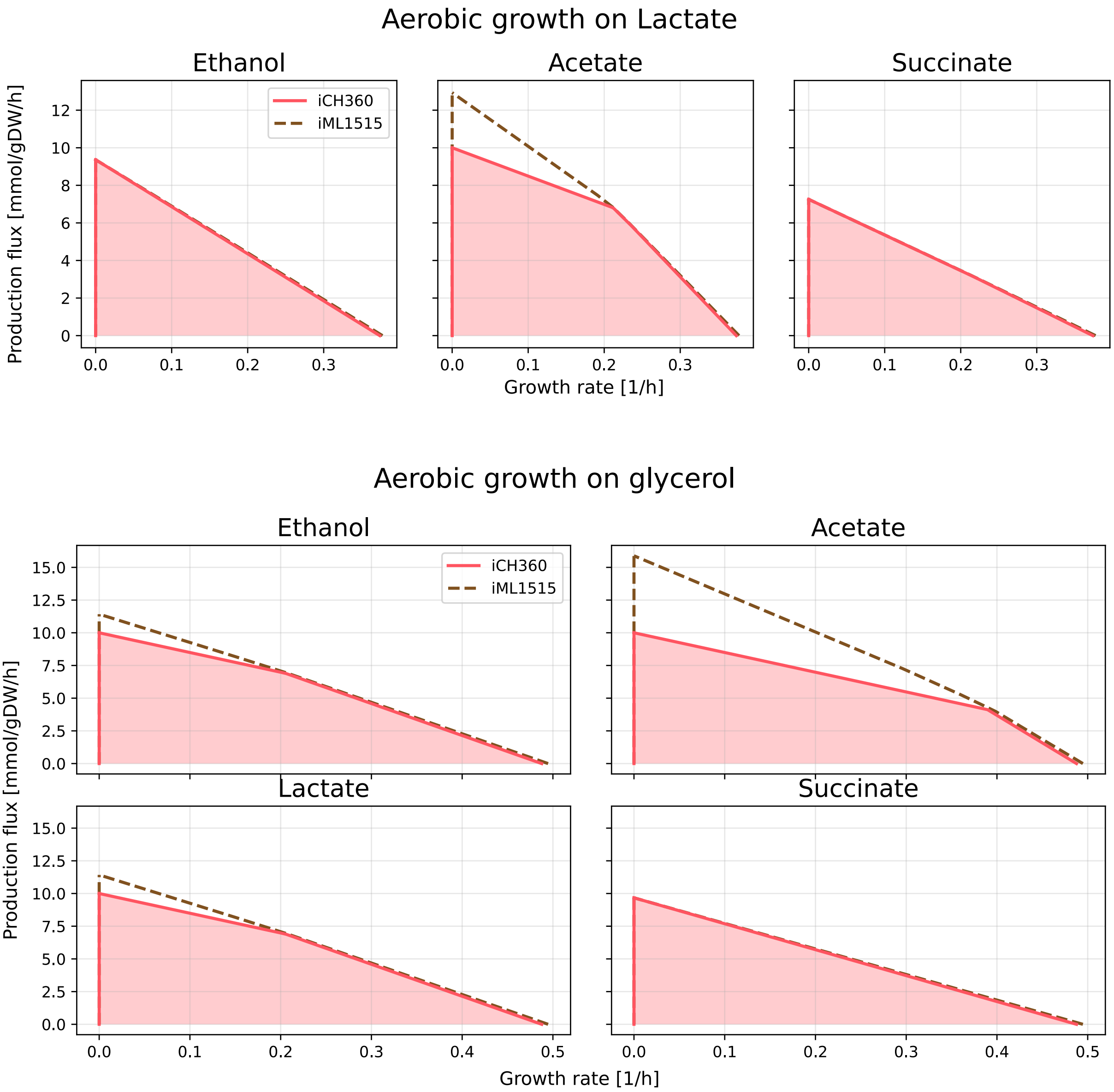

Fig H: Comparison of production envelopes between iCH360 and its parent model iML1515. Top: production of ethanol, acetate and succinate during aerobic growth on lactate. Bottom: production of ethanol, acetate, lactate and succinate during aerobic growth on glycerol. Note that the dashed line representing the production envelope of iML1515 is sometimes hidden behind the blue line corresponding to iCH360.

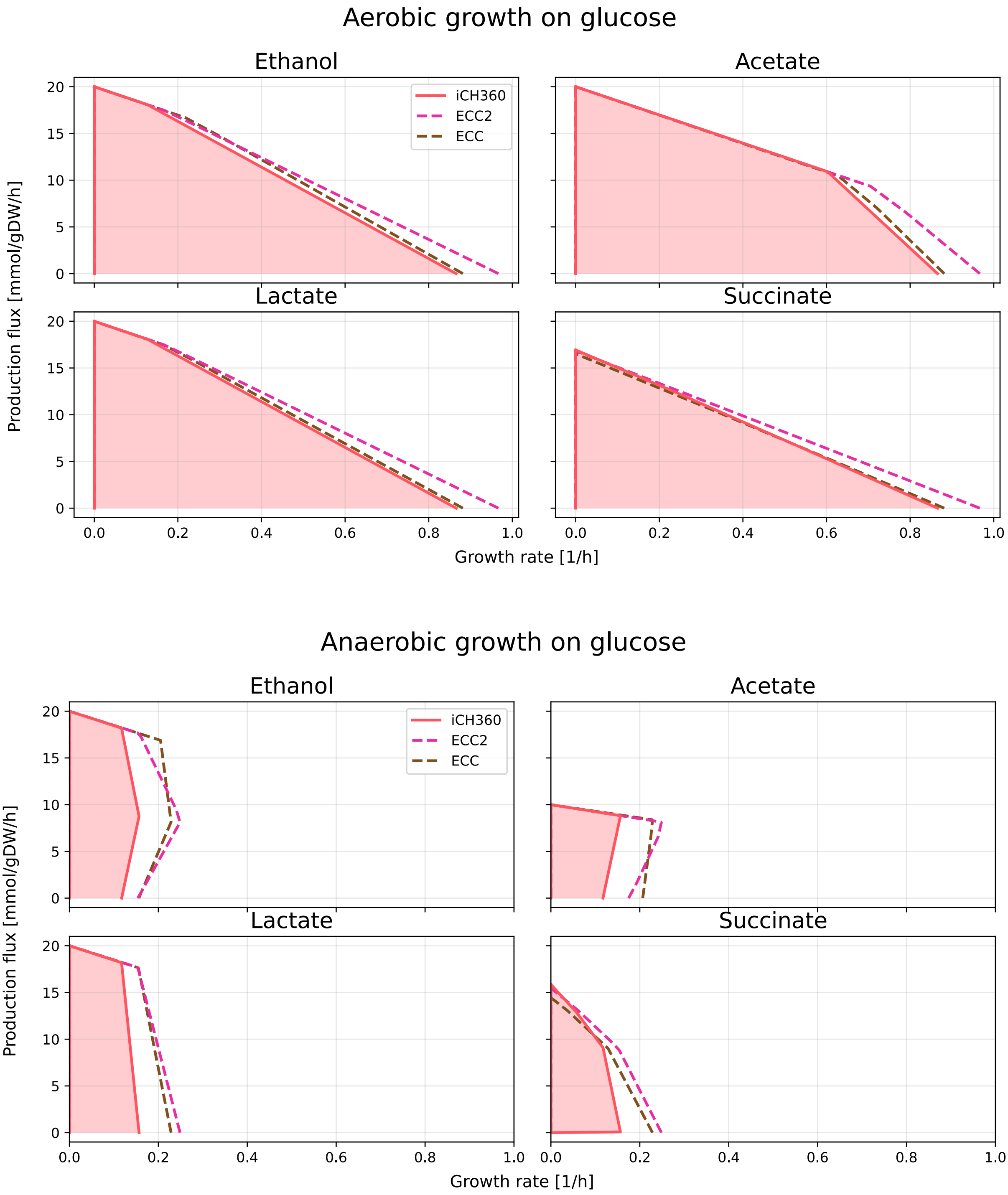

Fig I: Comparison of production envelopes between *i*CH360 and other medium-scale models, namely *E. coli* Core (ECC) and *E. coli* Core 2 (ECC2) for growth on glucose as a sole carbon source. Top: production of ethanol, acetate and succinate under aerobic conditions. Bottom: production of ethanol, acetate, lactate and succinate under anaerobic conditions. Additional comparisons between the three models are available in the repository supporting this manuscript.

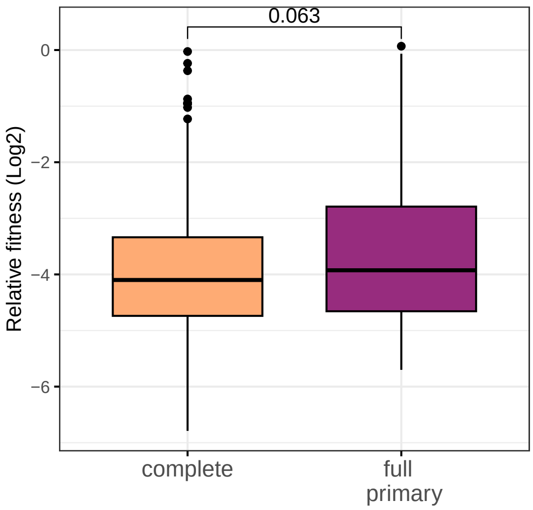

Fig J: Fitness losses associated with disruptions of catalytic edges. There is no significant difference between the fitness effects of disruptions classified as complete disruptions (disruption of all catalytic edges for a reaction) and full primary disruption (disruption of all primary catalytic associations, but with remaining secondary ones), according to a Wilcoxon rank-sum test, $p = 0.063$.

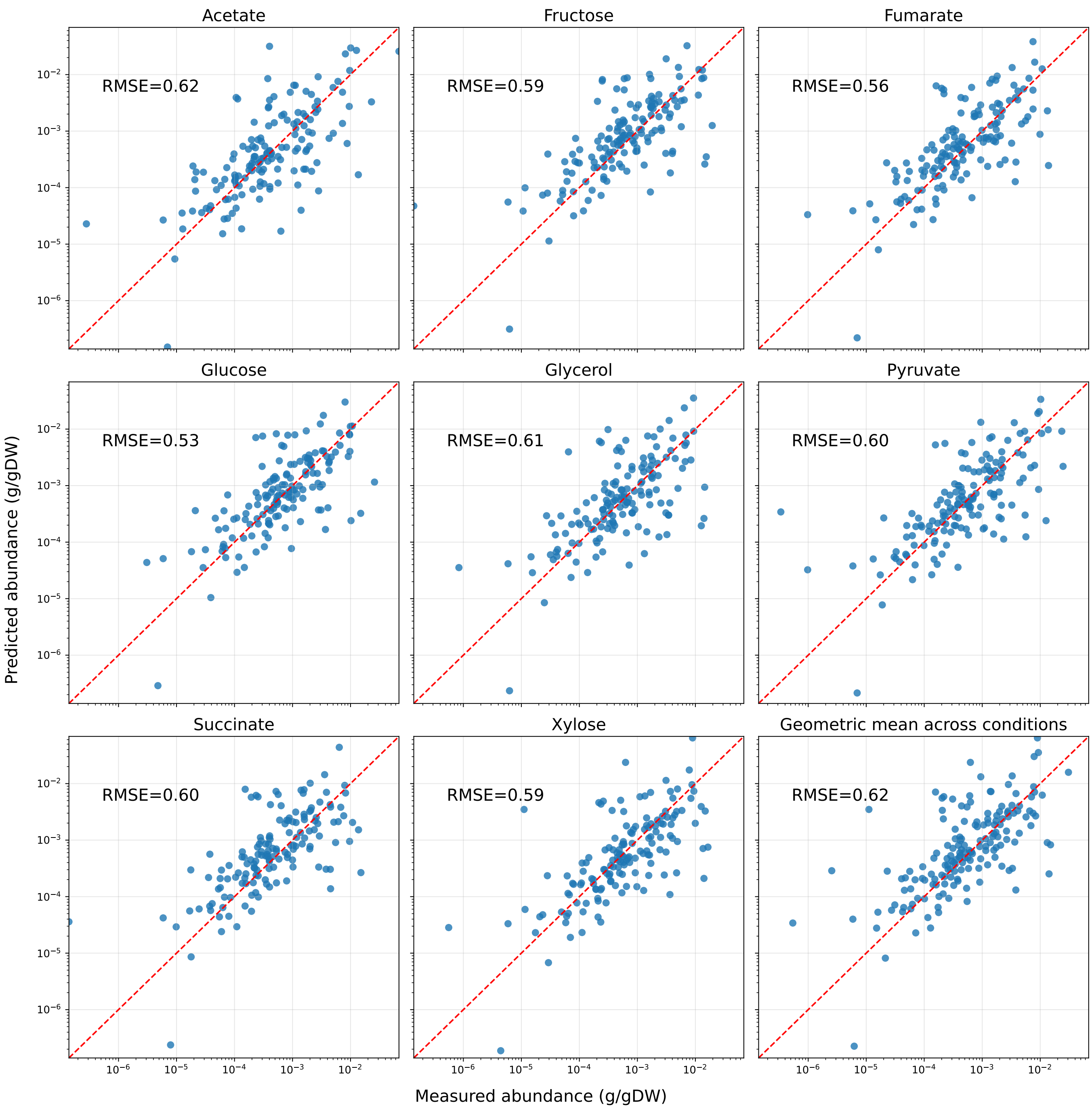

Fig K: Predicted proteome allocation across growth conditions. To obtain these predictions, we parametrised the model using the turnover parameter data set from Heckmann et al. (2020) [11]. The bottom-right panel shows the geometric means of measurements and predictions across conditions.

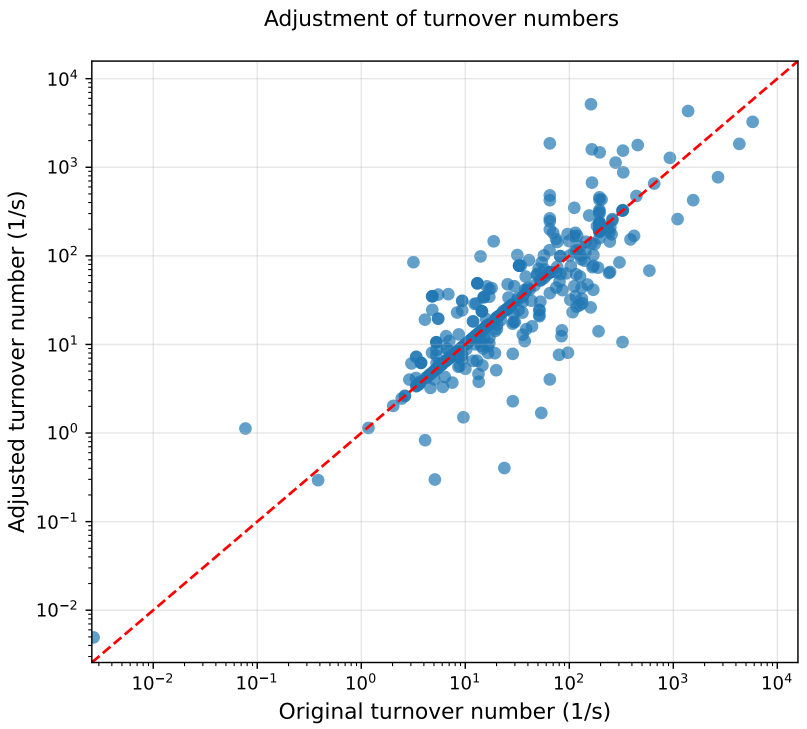

Fig L: Turnover parameters used in EC-iCH360 before and after performing the adjustment procedure (see Results and Methods in the main text, as well as Section F for details). The original parameter data set (plotted on the x-axis) was obtained from Heckmann et al. (2020) [11].

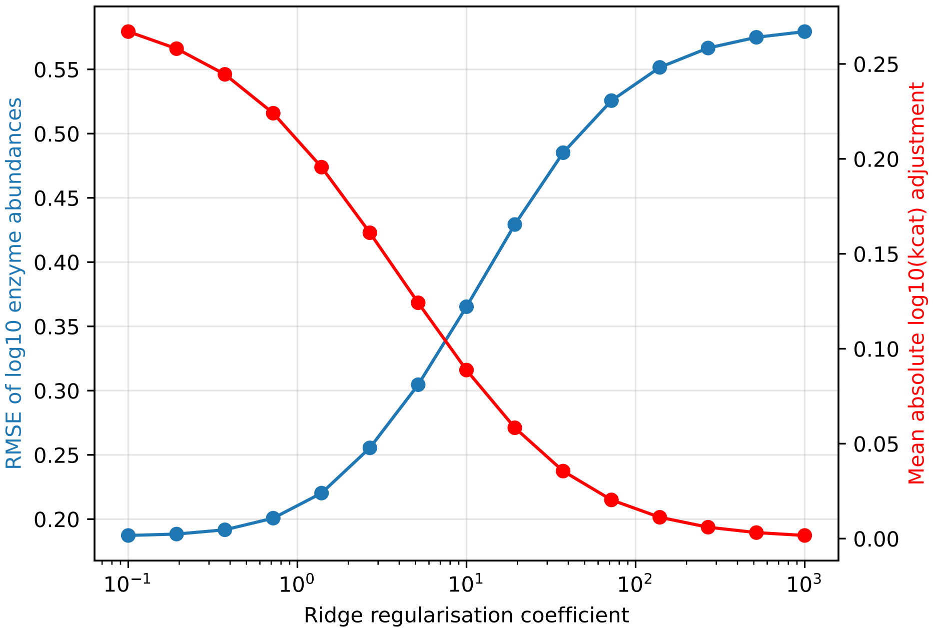

Fig M: Impact of the magnitude of the ridge regularisation coefficient (ρ in Eq. (22)) on the outcome of the adjustment procedure for turnover numbers. The blue curve represents the RMSE between measured and predicted log-enzyme abundances and decreases monotonically as less regularisation is applied to the problem. The red curve represents the mean absolute deviation between original and adjusted log-turnover numbers, which follows the opposite trend and converges to 0 as more regularisation is applied.

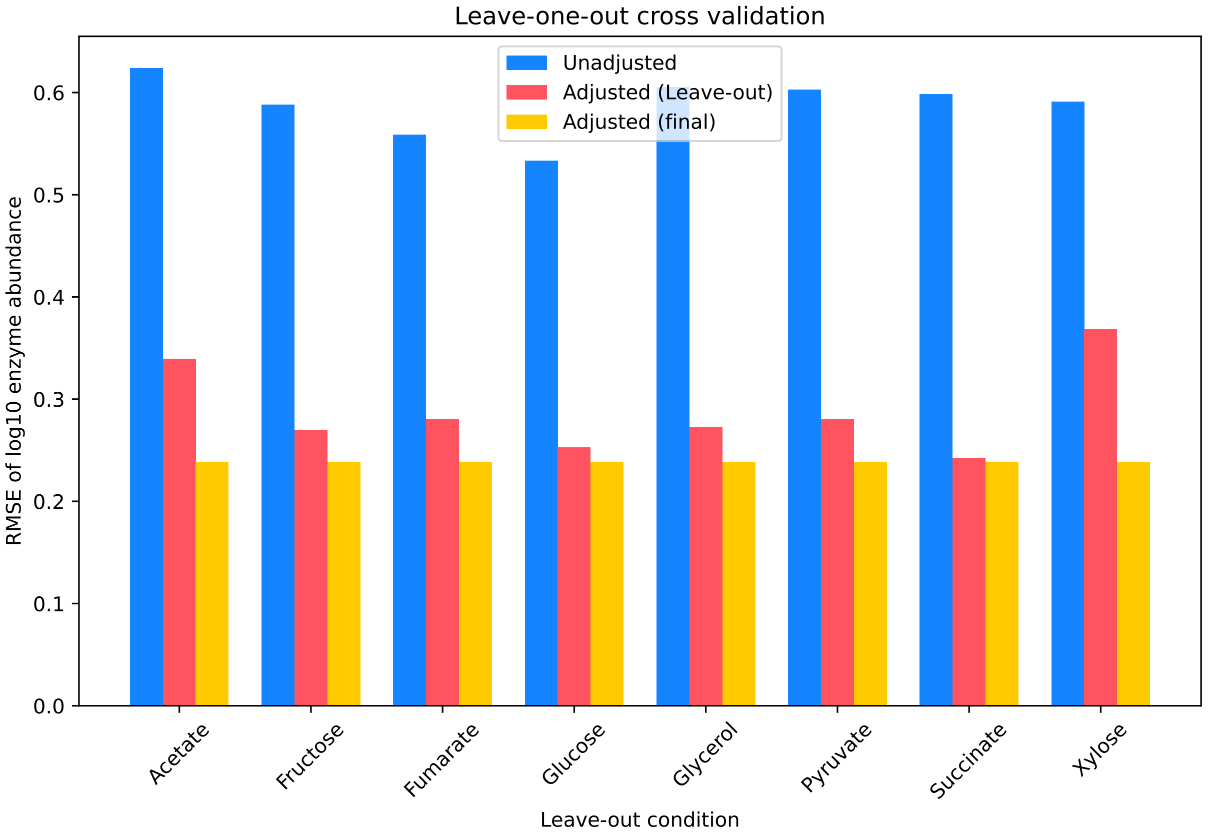

Fig N: Leave-one-out cross-validation for the turnover parameter adjustment procedure. For each condition in the dataset, the graph shows the RMSE (computed for $\log_{10}$-transformed values) between measurements and predictions of enzyme abundances in that condition. Blue bars show the RMSE computed using the initial, unadjusted parameter set from Heckmann et al. (2020) [11]. Red bars show the RMSE computed after parameter fitting, but excluding the condition for which the RMSE is evaluated from the training dataset. Finally, yellow bars show the RMSE computed using the final adjusted parameter set, obtained including all conditions in the training dataset.

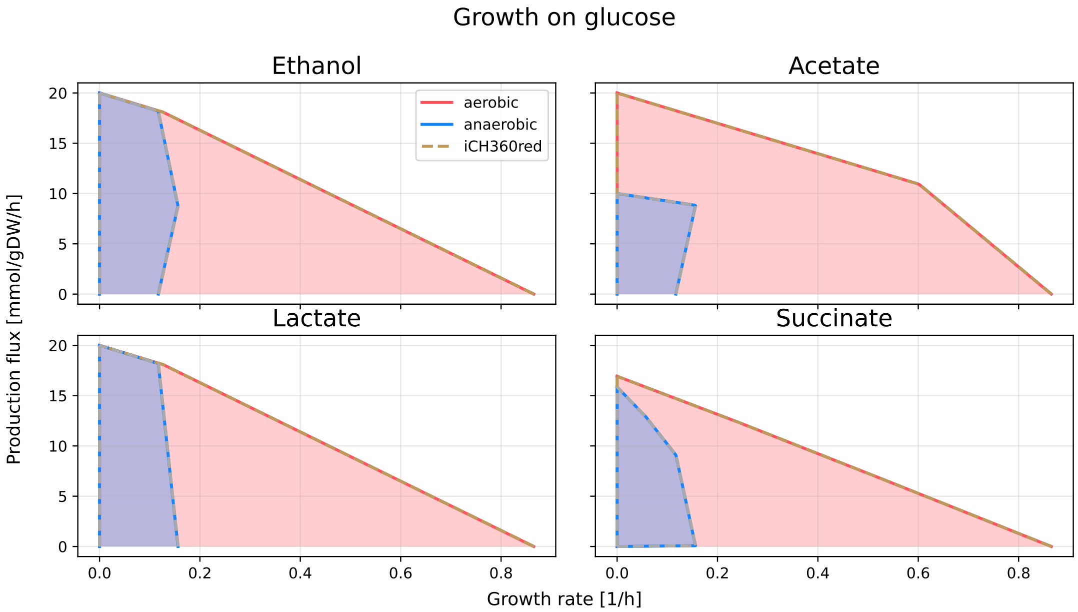

Fig O: Comparison of production envelopes for growth on glucose between iCH360 and the reduced variant $i\mathrm{CH360}_{\mathrm{red}}$ used for elementary flux mode enumeration and analysis. For the products and growth conditions shown, the two models have virtually identical solution spaces.

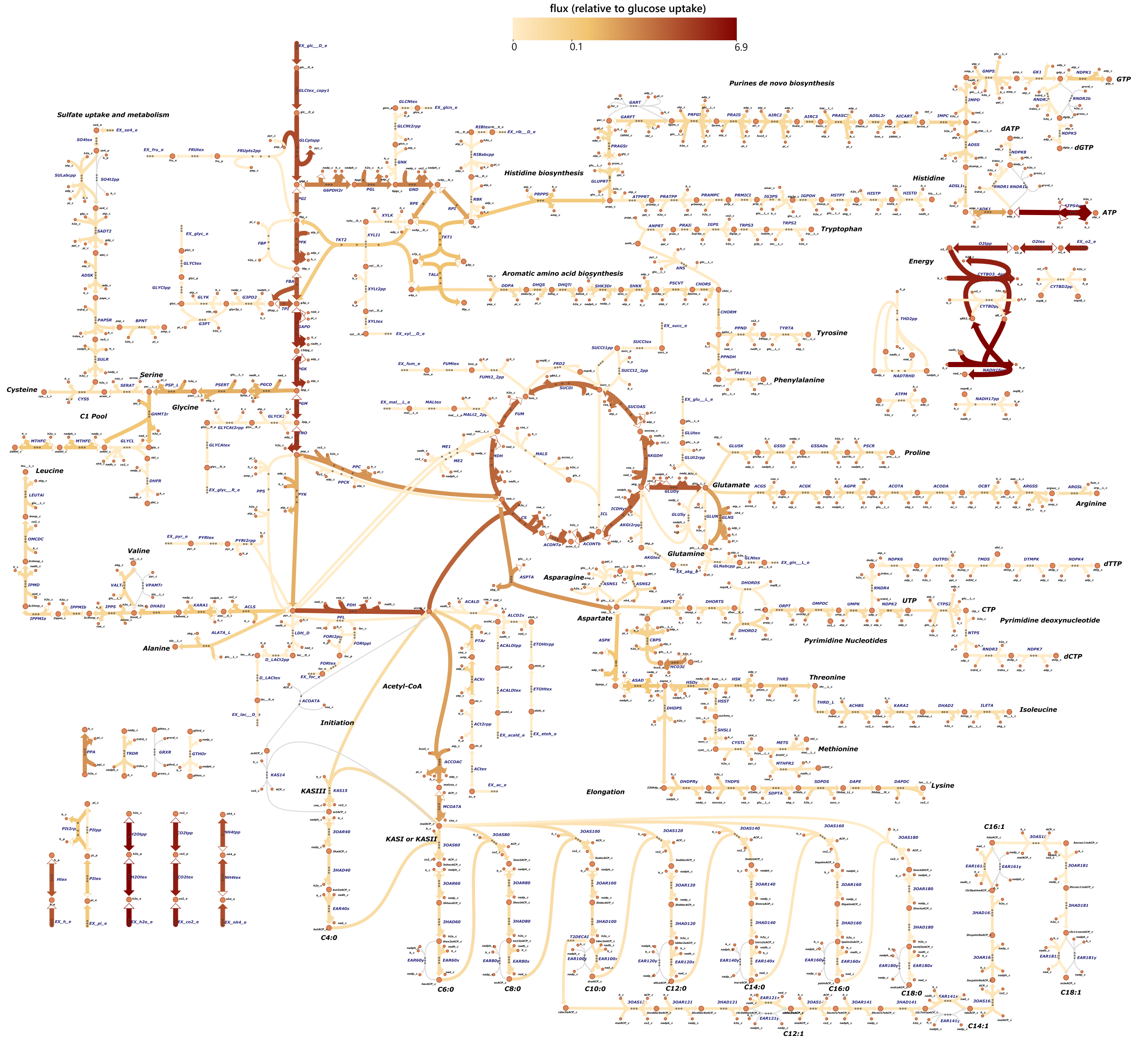

Fig P: Metabolic flux distribution corresponding to the maximum yield elementary flux mode (see main text). The mode is purely respiratory, with no excretion of typical fermentation by-products such as acetate, ethanol, or lactate. The graphics were produced in Escher [9]. Note that fluxes in the mode were normalised by the glucose uptake rate, and a nonlinear colormap was used for improved clarity.

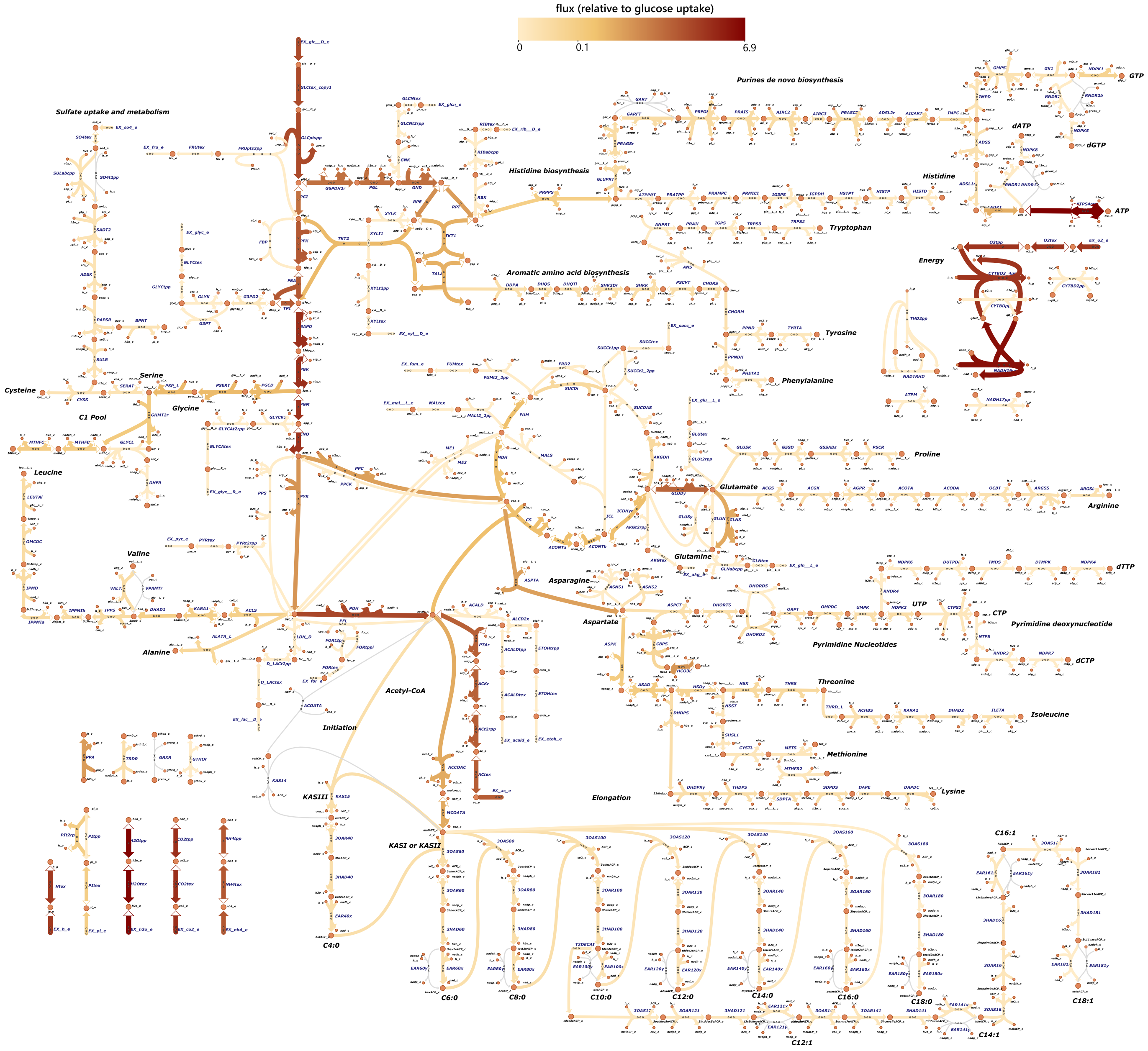

Fig Q: Metabolic flux distribution corresponding to the maximum growth elementary flux mode (see main text). The mode shows a mixed respiratory/fermentative phenotype, with significant acetate excretion. The graphic was produced in Escher [9]. Note that fluxes in the mode were normalised by the glucose uptake rate, and a nonlinear colormap was used for improved clarity.

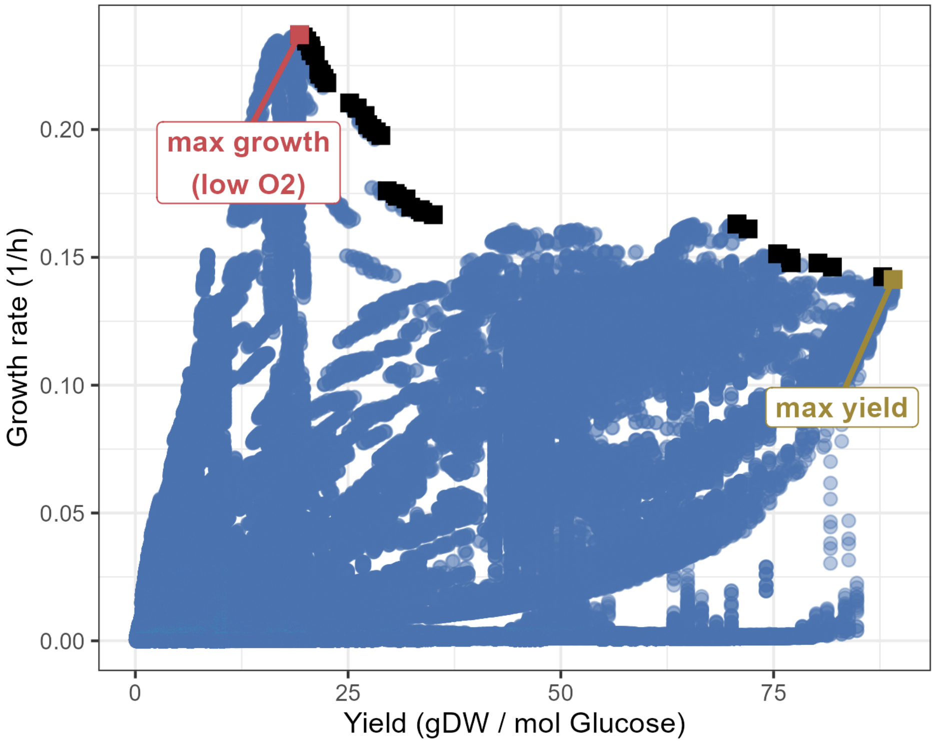

Fig R: EFM growth-yield trade-off front under simulated low oxygen conditions. The figure resembles Figure 5 in the main text, but was obtained by setting a 1000-fold higher cost for the cytochrome reactions. The higher enzyme cost is used to mimic the growth condition in which the oxygen-consuming reaction must operate at very low enzyme saturation, thus requiring higher enzyme-mass investment per unit flux. Black squares denote Pareto optimal EFMs. The Pareto front is much broader than the one obtained with the original enzyme cost for the oxygen-consuming reactions.

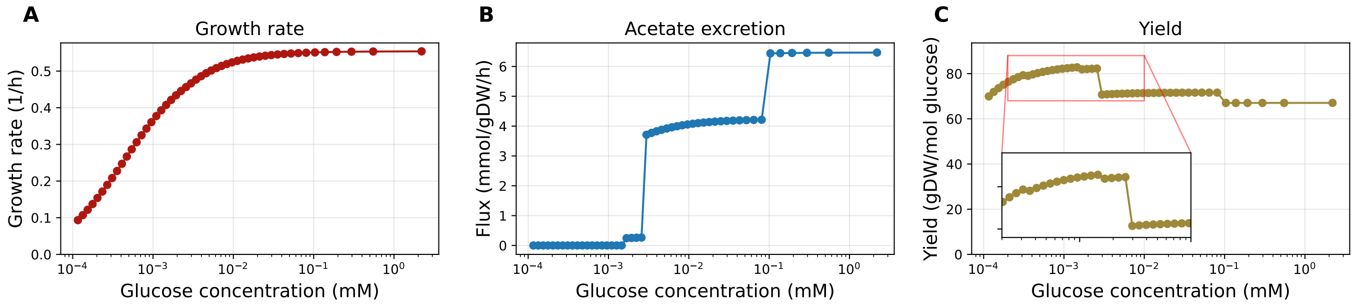

Fig S: satFBA results (corresponding to Figure 6 in the main text) obtained by enforcing a lower bound on the ATP maintenance flux equal to 6.86 mmol/gDW/h (taken directly from the parent model *i*ML1515). In this case, the optimal solution is no longer an EFM. This is evident from the yield profile (C), which is not piecewise constant with respect to the external glucose concentration.

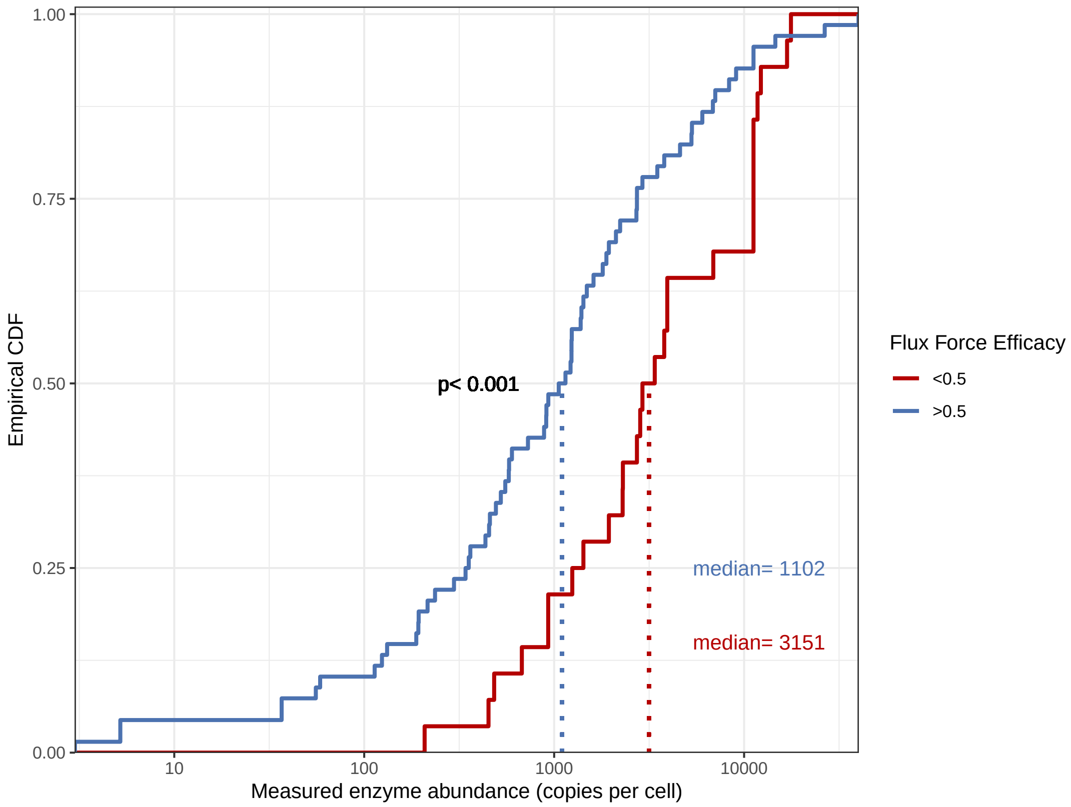

Fig T: Empirical cumulative distribution functions (CDF) of measured enzyme abundances for reactions with predicted flux force efficacies above or below 50% (blue and red curves, respectively). The two functions are significantly different ($p < 0.001$, two-sided Wilcoxon rank-sum test). The low-efficacy group shows an approximately 3-fold higher median enzyme abundance than the high efficacy group.

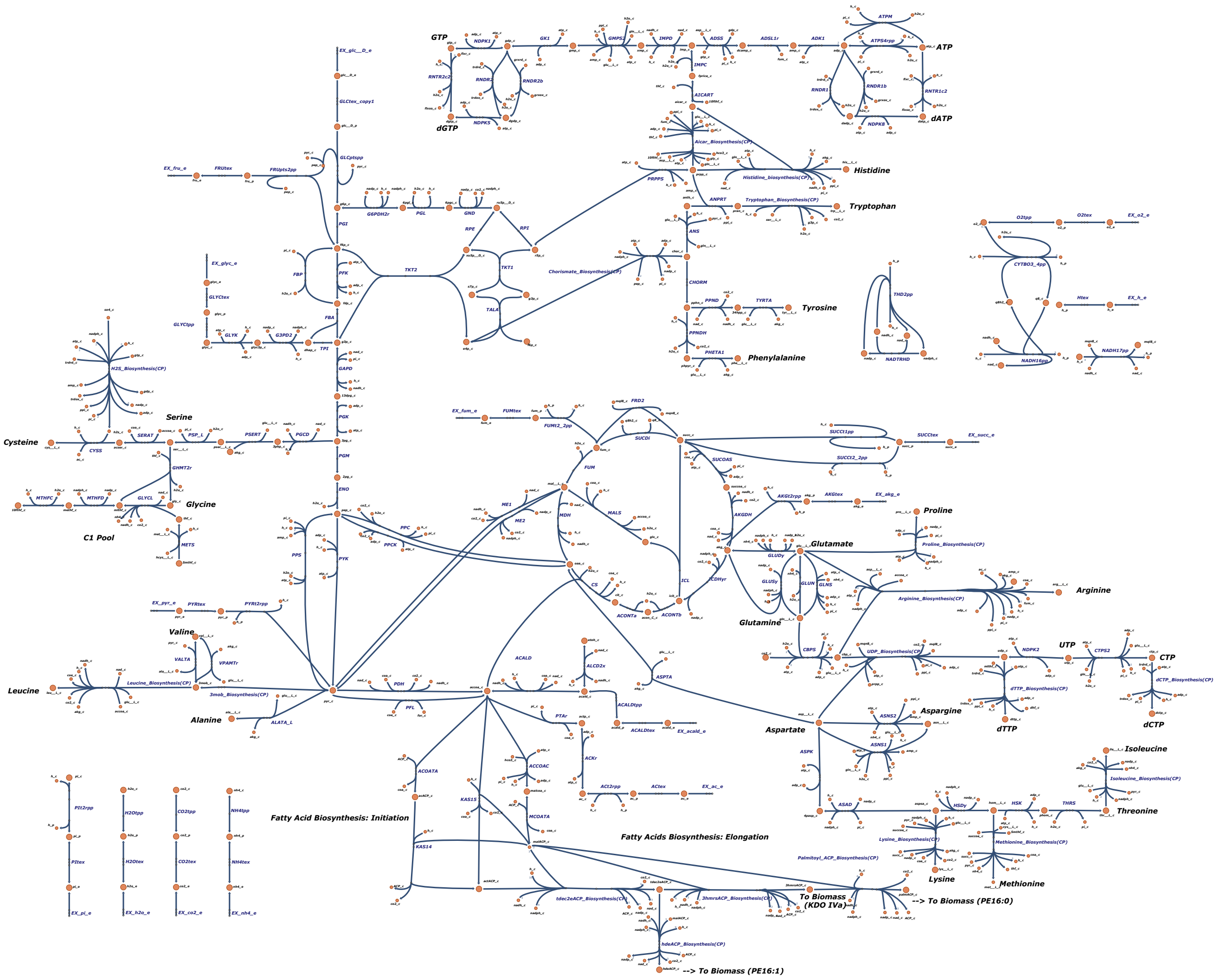

Fig U: Compressed metabolic map of *i*CH360, wherein linear pathways longer than two reactions were lumped into single effective reactions. The map was produced in Escher [9] and can be used to visualize fluxes, metabolite concentration, and gene expression data.

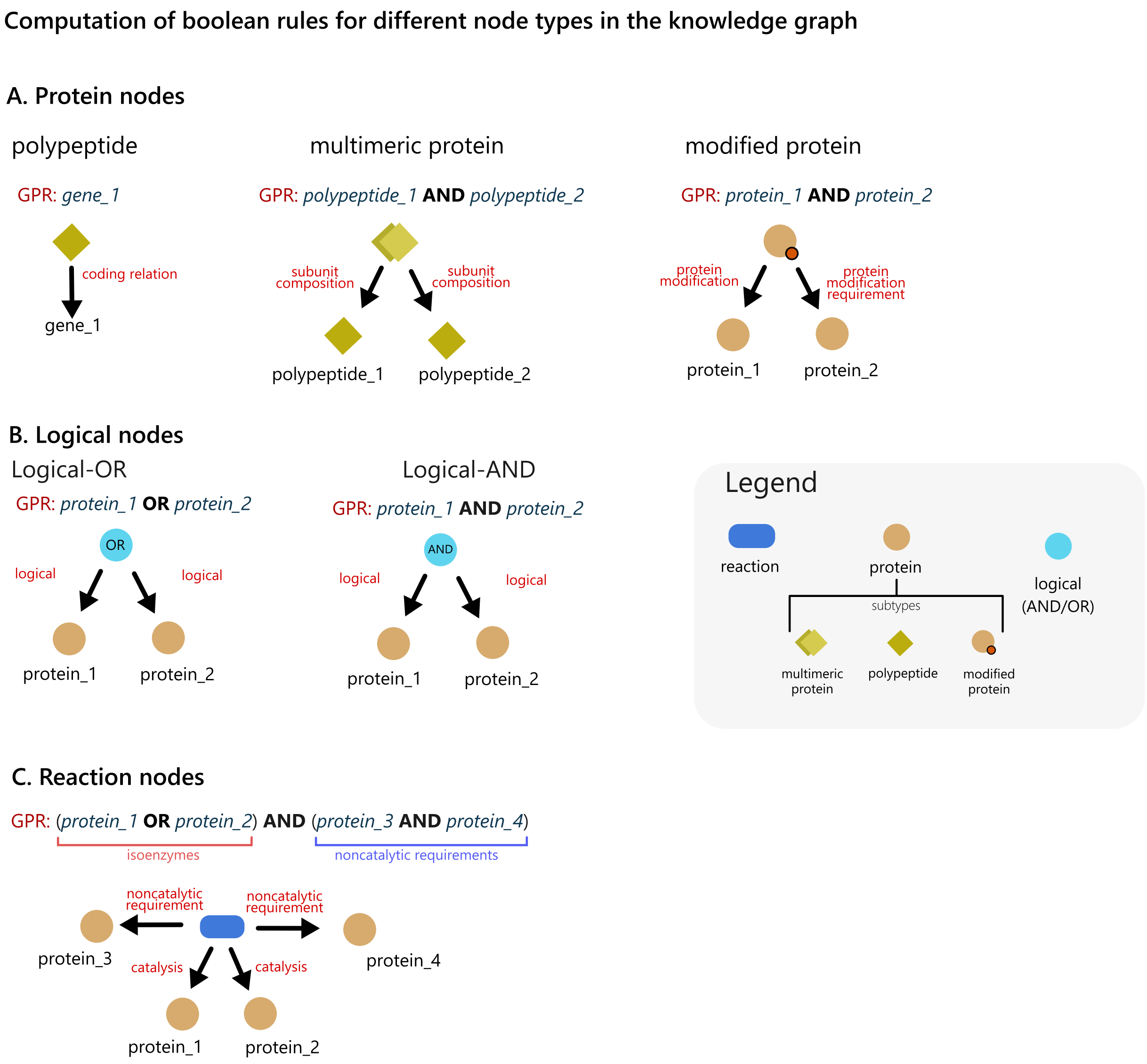

Fig V: Computation of Boolean gene-protein-reaction (GPR) rules with the knowledge graph. For each node in the graph (the top node in each diagram), a Boolean expression can be constructed to describe the state of a node (active or inactive) in terms of the state of its child nodes. The exact form of this Boolean expression depends both on the node type (e.g. protein, reaction, or logical) and on the type of edges that connect it with its neighbours (e.g. catalysis, subunit composition, etc.). Using these rules, the state of a reaction can be expressed, ultimately, solely in terms of genes in the model, as per convention in standard metabolic models. These GPR rules define a map between a genotype (set of active genes) to a phenotype (set of active reactions), and can be used to simulate *in silico* the effect of given gene knockouts.

Supplementary Tables

Table A: Some anecdotal examples of unrealistic predictions obtained when running FBA analyses on large-scale models (here, we considered the genome-scale parent of *i*CH360, *i*ML1515). We stress that these behaviors are not the result of errors in the large model. Rather, they result from applying simple methods with few constraints, such as FBA, to a large model with many degrees of freedom. Note that the examples shown here were not obtained through a thorough or systematic investigation of the model prediction abilities.

Example	Description
Production of fatty acids	In the (p)FBA solution computed on *i*ML1515 using glucose as a carbon source and growth as an objective, the canonical fatty acid production pathway in *E. coli* (see Fig C) is completely unused. Instead, the genome-scale model runs beta-oxidation in reverse to produce fatty acids.
Anaerobic pyruvate production	Under anaerobic conditions, *i*ML1515 can uptake external CO_2 and use it as a sink for glycolytic electrons, reducing acetyl-CoA produced by PFL back into pyruvate (see Section C). This behavior, which allows the model to channel additional carbon towards pyruvate, is thermodynamically unrealistic under ambient CO_2 conditions.
Pyruvate auxotrophic strain knock-outs prediction	To construct a pyruvate auxotrophic strain, only a few knock-outs in the central metabolism are necessary (*maeA*, *maeB*, *pck* [12]). However, *i*ML1515 uses reactions in amino acid degradations bypassing these knock-outs.
Acetyl-CoA auxotrophic strain knock-outs prediction	Similarly, knock-out of 4 genes (*aceEF*, *pflB*, *poxB*), disrupting PDH, PFL and POX reactions, respectively, results in an acetyl-CoA auxotrophic strain that is unable to grow on glucose as the sole carbon source [12]. These knockouts are bypassed *i*ML1515, which can use a number of additional pathways to produce acetyl-CoA (see Section C).

Table B: Description of node types in the knowledge graph supporting the stoichiometric model. The IDs used in the "Example" column refer to node identifiers in the graph.

Node type	Node subtype	Description	Example
reaction		A mass balanced chemical or biochemical reaction	bigg:GAPD
protein	polypeptide	A single polypeptide coded by a gene	GAPDH-A-MONOMER
	multimer	A complex formed by stoichiometric binding of different polypeptides and/or other complexes	GAPDH-A-CPLX
	modified protein	A polypeptide or multimer which underwent post-translational modification	LIPOYL-GCVH
gene		A gene	b1779
metabolite		An organic or inorganic molecule	L-ASPARTATE
logical-AND/ logical-OR		Used as an intermediate node to encode arbitrary logical rules in GPRs generated through the graph (e.g. one of two proteins being required by another node)	THIOREDOXINS

Table C: Classification and properties of edge types (and biological meaning of their associated weights, when applicable) in the *i*CH360 graph data structure supporting the stoichiometric model.

Edge type	Parent node type(s)	Child node type(s)	Subtype	Description	Example
catalysis	reaction	protein	primary	The default catalytic relationship between a reaction and an enzyme.	bigg:PFK → 6PFK-1-CPX
			secondary	A catalytic relationship between a reaction and an enzyme, where the enzyme had been shown in literature (based on *in vitro* or *in vivo* evidence) to account for only minor catalytic activity for the reaction when compared to another (primary) isozyme. Notes and references to the relevant literature for the secondary annotation are included as edge metadata.	bigg:PFK → 6PFK-2-CPX
			inactive	Indicates that the child protein is an enzyme for the parent reaction, but it's inactive in the K-12 strain due e.g. to a frameshift mutation. There are only two such edges in the model, and they both involve the same enzyme (Acetohydroxyacid synthase II) encoded by the *ilvG* and *ilvM* genes	bigg:ACHBS → ACETOLACTSYNII-CPLX
non-catalytic requirement	reaction	protein		Indicates that the child protein is required by the parent reaction, although not as a catalyst. Typical examples include proteins used as cofactors (e.g. glutaredoxins) in the reaction or featuring as prosthetic groups for a metabolite involved in the reaction (e.g. Acyl-Carrier-protein)	bigg:ACOATA → ACP-MONOMER
subunit composition	protein	protein	requirement	Indicates that the child node is a subunit of the parent node and is required for the correct functioning of the complex. The weight of the edge indicates the stoichiometry of the subunit in the complex.	FABA-CPLX $\xrightarrow{2}$ FABA-MONOMER
			accessory	Indicates that the child protein is an accessory subunit of the parent protein, meaning it can be part of the complex (potentially enhancing or modulating its function), but it's not strictly required for the complex to perform its physiological function. The weight of the edge indicates the stoichiometry of the subunit in the complex.	ATPSYN-CPLX $\xrightarrow{1}$ EG10106-MONOMER
protein modification	protein	protein		Indicates that the parent protein is a obtained by post-translational modification of the child protein.	PYRUVFORMLY-CPLX → PYRUVFORMLY-INACTIVE-CPLX
protein modification requirement	protein (modified-protein)	protein		Indicates that the child protein is required to accomplish the post-translational modification leading to the parent protein.	PYRUVFORMLY-CPLX → PFLACTENZ-MONOMER
coding relation	protein	gene		Indicates that the child gene codes for the parent polypeptide	RIBOKIN-MONOMER → b3752
regulation	protein	metabolite, protein		Indicates that the child metabolite or protein is a regulator for the enzyme. Information about the regulation mode (activation vs inhibition), the regulation mechanism (competitive vs allosteric) and the regulated reaction (if the enzyme catalyses multiple) is provided as edge metadata whenever available. If present, the weight of the edge denotes the activation/inhibition constant for the interaction as reported in EcoCyc, with units indicated as edge metadata.	SHIKIMATE $\xrightarrow{160.0\mu M}$ AROE-MONOMER
putative association	reaction	protein		Indicates that a putative association between the reaction and the protein has been proposed in literature.	bigg:PFL → EG11910-MONOMER
logical	logical AND/OR	any		Connects logical operator nodes to downstream nodes. Used to create arbitrary complex logic relations in the graph.	THIOREDOXINS<br>→ RED-THIOREDOXIN-MONOMER<br>→ RED-THIOREDOXIN2-MONOMER<br>(In this example, the logical edges are used to indicate that the THIOREDOXINS node (of type logical-OR) is active when any of the two child nodes (representing two thioredoxins found in *E. coli*) are active.

Table D: Manual curation of the reaction pruning process used to construct iCH360$_{\text{red}}$. Each reaction set represents a set of alternative routes for the production of the same metabolite (but using, for example, different cofactors). For each set, the most physiologically relevant option, based on available literature whenever available, was preserved in the reduced model variant.

Reaction set	Pruned in iCH360red	Notes
EAR(n)x, EAR(n)y *	EAR(n)y	Enzyme FabI can work with both NADH/NADPH, but higher activity was found with NADH [13]
ACOATA, KAS14, KAS15	ACOATA, KAS14	Initiation of fatty acid biosynthesis can occur by either direct condensation of acetyl-CoA with malonyl-ACP (KAS15) or by transacylation of acetyl-CoA followed by condensation with malonyl-ACP (ACOATA + KAS15). Because the transacylacylase activity of FabH (ACOATA) has been to be significantly lower than its condensation activity (KAS15) [14], only the former pathway is maintained in iCH360red.
VALTA, VPAMTr	VPAMTr	These are both routes to production of valine. We keep VALTA (*ilvE*) as it is the last step in the canonical valine biosynthesis route.
RNDR1, RNDR2, RNDR1b, RNDR2b	RNDR1b, RNDR2b	The ribonucleoside diphosphate reductase can work both with the thioredoxin and glutaredoxin redox systems. iCH360 retains only the thioredoxin version.
SULabcpp, SO4t2pp	SO4t2pp	SULabcpp is an ATP-mediated active transport of sulfate in the cell via an ATP-binding-cassette (ABC) transporter [15], while SO42tpp (*cysZ*) is a proton symporter [16]. We maintain the former as its impairment was shown to lead to cysteine auxotrophy [15].

* $n \in (60, 80, 100, 120, 140, 160, 180, 121, 141, 161, 181)$

Table E: Numbers of elementary flux modes enumerated for the reduced model variant *i*CH360red under different growth conditions. Numbers in brackets represent the number of EFMs after filtering. Filtered modes include only those supporting biomass flux and, for aerobic conditions, those that a) have non-zero oxygen uptake and b) do not use either of three reactions (PFL, DHORD5, FRD2), which are known to be only physiologically active under anaerobic conditions.

	number of EFMs (filtered)	
	Aerobic	Anaerobic
Glucose	13468719 (1035696)	204028 (195670)
Pyruvate	1763631 (135266)	6949 (6480)
Glycerol	922217 (82112)	NA
Acetate	38099 (7596)	NA
Lactate	1270315 (5897)	1497 (1424)

References

[1] Bizouarn T, van Boxel GI, Bhakta T, Jackson JB. Nucleotide binding affinities of the intact proton-translocating transhydrogenase from Escherichia coli. Biochimica et Biophysica Acta (BBA) - Bioenergetics. 2005;1708(3):404–410. doi:10.1016/j.bbabio.2005.04.004.

[2] Zelcbuch L, Razo-Mejia M, Herz E, Yahav S, Antonovsky N, Kroytoro H, et al. An in vivo metabolic approach for deciphering the product specificity of glycerate kinase proves that both E. coli's glycerate kinases generate 2-phosphoglycerate. PLOS ONE. 2015;10(3):e0122957. doi:10.1371/journal.pone.0122957.

[3] Bartsch O, Hagemann M, Bauwe H. Only plant-type (GLYK) glycerate kinases produce d-glycerate 3-phosphate. FEBS Letters. 2008;582(20):3025–3028. doi:10.1016/j.febslet.2008.07.038.

[4] He H, Höper R, Dodenhöft M, Marlière P, Bar-Even A. An optimized methanol assimilation pathway relying on promiscuous formaldehyde-condensing aldolases in E. coli. Metabolic Engineering. 2020;60:1–13. doi:10.1016/j.ymben.2020.03.002.

[5] Krivoruchko A, Zhang Y, Siewers V, Chen Y, Nielsen J. Microbial acetyl-CoA metabolism and metabolic engineering. Metabolic Engineering. 2015;28:28–42. doi:10.1016/j.ymben.2014.11.009.

[6] Zhang S, Yang W, Chen H, Liu B, Lin B, Tao Y. Metabolic engineering for efficient supply of acetyl-CoA from different carbon sources in Escherichia coli. Microbial Cell Factories. 2019;18(1):130. doi:10.1186/s12934-019-1177-y.

[7] Bekiaris PS, Klamt S. Automatic construction of metabolic models with enzyme constraints. BMC Bioinformatics. 2020;21(1):19. doi:10.1186/s12859-019-3329-9.

[8] Maranas CD, Zomorrodi AR. Optimization methods in metabolic networks. Wiley; 2016.

[9] King ZA, Dräger A, Ebrahim A, Sonnenschein N, Lewis NE, Palsson BO. Escher: A web application for building, sharing, and embedding data-rich visualizations of biological pathways. PLOS Computational Biology. 2015;11(8):e1004321. doi:10.1371/journal.pcbi.1004321.

[10] Hädicke O, Klamt S. EColiCore2: a reference network model of the central metabolism of Escherichia coli and relationships to its genome-scale parent model. Scientific Reports. 2017;7(1):39647. doi:10.1038/srep39647.

[11] Heckmann D, Campeau A, Lloyd CJ, Phaneuf PV, Hefner Y, Carrillo-Terrazas M, et al. Kinetic profiling of metabolic specialists demonstrates stability and consistency of in vivo enzyme turnover numbers. Proceedings of the National Academy of Sciences. 2020;117(37):23182–23190. doi:10.1073/pnas.2001562117.

[12] Yu H, Li X, Duchoud F, Chuang DS, Liao JC. Augmenting the Calvin-Benson-Bassham cycle by a synthetic malyl-CoA-glycerate carbon fixation pathway. Nature communications. 2018;9(1):2008. doi:10.1038/s41467-018-04417-z.

[13] Bergler H, Fuchsbichler S, Högenauer G, Turnowsky F. The enoyl-[acyl-carrier-protein] reductase (FabI) of Escherichia coli, which catalyses a key regulatory step in fatty acid biosynthesis, accepts NADH and NADPH as cofactors and is inhibited by palmitoyl-CoA. European Journal of Biochemistry. 1996;242(3):689–694. doi:10.1111/j.1432-1033.1996.0689r.x.

[14] Tsay JT, Oh W, Larson TJ, Jackowski S, Rock CO. Isolation and characterization of the beta-ketoacyl-acyl carrier protein synthase III gene (fabH) from Escherichia coli K-12. Journal of Biological Chemistry. 1992;267(10):6807–6814. doi:10.1016/S0021-9258(19)50498-7.

[15] Sirko A, Zatyka M, Sadowy E, Hulanicka D. Sulfate and thiosulfate transport in Escherichia coli K-12: evidence for a functional overlapping of sulfate- and thiosulfate-binding proteins. Journal of Bacteriology. 1995;177(14):4134–4136.

[16] Zhang L, Jiang W, Nan J, Almqvist J, Huang Y. The Escherichia coli CysZ is a pH dependent sulfate transporter that can be inhibited by sulfite. Biochimica et Biophysica Acta (BBA) - Biomembranes. 2014;1838(7):1809–1816. doi:10.1016/j.bbamem.2014.03.003.
