# Supplementary material for: A compact model of Escherichia coli core and biosynthetic metabolism
Source: PLoS Comput Biol. 2025 Oct 13;21(10):e1013564. doi: 10.1371/journal.pcbi.1013564 (PMC12533975; doi:10.1371/journal.pcbi.1013564)
Supplement: Attachment — Submitted filename: GMG_review_v2.pdf [file pcbi.1013564.s003.pdf]

This is an elegant and well-written paper that presents an expertly curated, medium-scale metabolic model of Escherichia coli. The quality of the work is impressive, and the resulting model will undoubtedly support numerous future applications.

Major-importance comments

- The catalytic disruption analysis and the associated interpretations need to be clarified (see the associated comments below).

Medium-importance comments

- Lines 161-171: You don't detail the **KEGG annotation**. It would be interesting for the reader to know a bit more about it here (as you display some information about it on figure 3A)

- Lines 203-219 and Method's section lines 505-518: **the catalytic disruption analysis is not clear to me**. If I understand correctly, you identify essential reactions for a given condition. Then, for each essential reaction, you individually knock-out the genes associated with it, and "propagate" the KO across the knowledge graph accordingly using the Boolean GPR rules you defined. You then "*catalogue the KO according to the reaction-level disruption it caused*" (and if the KO affects multiple essential reaction, you catalogue it using the strongest disruption). Finally, you assign a fitness for each KO-disruption category-condition tuple. If I am right and this is what you do, then I believe your method section is not super clear. I would suggest the following clarifications, or something similar:

  <u>Suggested clarification for results section</u>: "*For each growth condition, we identified essential reactions by simulating in silico knockouts of reactions and selecting those that abolish growth. For each such reaction, we identified all catalyzing genes via our knowledge graph. We then performed simulated knockouts for each gene, propagated the disruption through the graph using Boolean GPR logic, and assessed the impact on the reaction(s). Based on which catalytic edges were lost, we assigned a disruption category to the KO as follows: (i) complete (all catalytic edges lost), (ii) full primary (only primary edges lost), (iii) partial primary (some primary edges lost), or (iv) secondary (only secondary edges lost). If a single gene knockout disrupted multiple reactions, we assigned the most severe disruption among them. These gene-condition-disruption tuples*

were then mapped to corresponding experimental fitness values from the Price et al. (2018)."

<u>Suggested clarification for methods section</u>: "*To simulate catalytic disruptions, we first identified condition-specific essential reactions using growth simulations. For each essential reaction, we enumerated associated genes and simulated their individual knockouts. Knockouts were propagated across the knowledge graph using Boolean logic rules, deactivating proteins and reactions as appropriate. Each resulting gene-condition pair was labeled with a disruption class depending on the type of enzyme-reaction associations lost: complete, full primary, partial primary, or secondary. If a gene knockout affected multiple reactions, we labeled the gene with the most severe disruption across all affected reactions. Finally, we compared predicted disruption types to measured fitness values from Price et al. (2018), using Wilcoxon rank-sum tests to assess statistical differences in fitness between disruption classes*."

Even if I get the analysis correctly, a strong limitation is that you do not simulate the weaker effect of secondary catalysis by using e.g. lower values for reaction boundaries in the GEM model. This should be discussed and balance your statement lines 218-219.

- L304-328 (Saturation FBA): Why did you only perform satFBA for glucose? Could you do it/would it be interesting to do it for other substrates (modulo the calibration of the Km values)?

- Line 507: "*a small number of known false positives*": **please provide the list** as supplementary

- Table 3: Please define Kmax_app and Kapp, and add their units

- Would it make sense to obtain a MEMOTE score of the model?

Minor-importance comments

- L13-14: "*We enriched the stoichiometric network with extensive biological information and quantitative data, enhancing the scope and applicability of the model*" → You could add few examples of these biological information and

quantitative data for the reader in the abstract here: "[...] *quantitative data (e.g., such as thermodynamics, kinetic constants, etc.)"*

- L38: And how many metabolites? Please add this information
- Fig 1: "*The map was created the metabolic*" → "with" is missing
- Line 109-110: please provide an estimated value (e.g. average percentage difference over all conditions) of the differences between the models. Why don't you test/show all the carbon sources that the models have in common?
- Line 121: "yield": please define what yield you refer to here (as different definitions of yield can be used, and it can be confusing for the reader). Also, please specify how the reader can infer the yield from the production envelope (for better clarity).
- Line 137 and Supplementary figure S7: "B" is missing
- Line 172: "estensive" → please correct
- Line 226: "(*Section)"* → please correct
- Figure S16 and S17: "*(Section)"* → please correct. Also, you could use a more contrasted color scale for better clarity
