# Supplementary material for: A compact model of Escherichia coli core and biosynthetic metabolism
Source: PLoS Comput Biol. 2025 Oct 13;21(10):e1013564. doi: 10.1371/journal.pcbi.1013564 (PMC12533975; doi:10.1371/journal.pcbi.1013564)
Supplement: Attachment — Submitted filename: iCH360 PCB Response to Reviewers.pdf [file pcbi.1013564.s004.pdf]

PCOMPBIOL-D-25-01048: Answers to reviewers' comments

Note: all line numbers referenced in our answers refer to the clean revised version (without tracked changes) of the manuscript

Reviewer #1

Genome-scale models of microbial metabolism offer a wide range of applicability from understanding to engineering the metabolism. However, the large size of these models can hinder its interpretability and visualization. To address these issues, authors had developed a compact model of Escherichia coli K-12 MG1655 (iCH360) extracted from the existing reconstruction, iML1515. One of the key contributions of the paper include enriching the network with a weighted knowledge graph constituting the reactions, proteins, genes and compounds as nodes and annotation of the catalytic relationship as either primary or secondary that together improved the gene essentiality predictions. Furthermore, development the escher maps can help the modellers visualize the flux distribution across a wide range of environmental conditions. Extensive computational experiments done on iCH360, shows its alignment and distinction compared to its parent model, iML1515. Overall, the compact model of E.coli is a valuable tool to the modelling community. However, this manuscript itself might benefit from a few modifications suggested below.

We thank the reviewer for the positive outlook on the manuscript.

Major comments:

1) Can the authors explain the reason for choosing Ethanol, Acetate, Lactate and Succinate in the production envelop analysis? How will the results change if any other products are chosen for this analysis?

The choice of these four products for the production envelopes comparison was based on their biotechnological relevance and is consistent with the products shown in the *E. coli* Core 2 work by Hadicke and Klamt (2017) [https://doi.org/10.1038/srep39647]. Additionally, the production envelopes for pyruvate are also shown to the reader in the context of the investigation of the acetate production differences between iCH360 and iML1515 (Supplementary Figure S7). We believe that these five metabolites and the three growth conditions shown cover a wide enough sample of typical use cases to appear in the manuscript. However, we have now provided the interested reader with an extended set of production envelopes comparisons focusing on 13 key metabolic precursors relevant to biotechnological applications (DOI: 10.1016/j.cell.2016.02.004). This is available in the repository supporting the paper at:

https://github.com/marco-corrao/iCH360_paper/blob/main/Manuscript_Figures/notebooks/additional_production_envelopes.ipynb

The results still support our conclusion that *i*CH360 is similar, but more precise than *i*ML1515. We now note in the caption for Fig.2 that “an extended set of production envelope comparisons between the models is available online in the code repository supporting this manuscript.”

2) In computing equivalent biomass reaction, does the coefficient of the biomass reaction vary based on alternate solutions that can be obtained in FBA (Multiple solutions possible for v*). Authors have mentioned that the biomass reaction will remain unique across the conditions. Can they shed more light on this? Has not even a single coefficient has changed across the conditions? If changed can the authors quantify it across the conditions and alternate solutions possible in FBA.

The equivalent biomass reaction was computed by extending the model with a set of manually curated peripheral pathways producing complex biomass requirements (e.g. phospholipids, lipopolysaccharides, murein, and others) from precursors in the model. While the FBA solution computed on this extended model (v* ref in Eq. 1, Section 2 in S1 Text) is indeed not unique, we observed no flux variability for the reactions in these curated pathways across all carbon sources supported by the model. Since the cost of complex biomass components in terms of model precursors depends on these additional reactions, the conversion is unchanged by the choice of growth conditions. Clearly, this is not true in general. If the additional pathway used to extend the model allowed for alternative conversion stoichiometries between model precursors and biomass components, the resulting equivalent biomass reaction would not be unique and would depend on the specific choice of reference distribution between these alternatives. More broadly, we note that biomass and maintenance reactions of other metabolic models also only represent an "average" of real world physiological states of the organism.

3) At line-507 the authors mention “A small number of known false positives, which are essential in iCH360 only due to its lack of certain reactions or pathways, were excluded from the analysis.” Can the authors explain how they got these false positives? In addition, list them out?

These refer to 4 genes, mapping to two reactions, namely the glycine cleavage system (GLYCL) and Thioredoxin reductase (TRDR). GLYCL is essential in *i*CH360 since we chose not to include a THF charging reaction, FTHFLi, present in *i*ML1515 (with empty “gene-reaction-rule”), since an enzyme specific for this activity is, to our knowledge, not present in *E. coli*. TRDR is essential in our model because a number of pathways using glutaredoxin as a cofactor are not included, making thioredoxin regeneration essential in our model, but not in practice. The false-essentiality of these reactions, which was known anecdotally, would only be relevant to very specific modelling scenarios. We have included this information in our Methods (L583-589) for the interested reader.

4) Authors have used pFBA solutions to calculate the MDF and report that all the flux distribution have

positive MDF highlighting their thermodynamic feasibility. However using pFBA will always remove loops in the flux distribution (Refer: https://academic.oup.com/bioinformatics/article/31/13/2159/195895) given that the objective function does not participate in any infeasible cycles. Can the authors shed some light on how the MDF value change on using FBA? Also, is it even possible to have a negative value for MDF on using pFBA?

While thermodynamically infeasible cycles are one possible cause of thermodynamic infeasibility in a network (and are indeed excluded from pFBA solutions), here we focused on the issue of thermodynamic feasibility under physiologically plausible metabolite concentration ranges, which is not guaranteed even in the absence of infeasible cycles (see e.g. https://doi.org/10.48550/arXiv.1803.04999 for more details). To avoid confusion, this aspect has been clarified in the revised manuscript (L380-381), where we now state "we first tested whether some typical flux distributions obtained from the model are thermodynamically feasible under realistic metabolite concentration ranges". In this sense, there is no general guarantee that a pFBA solution has a positive MDF (i.e. is thermodynamically feasible given the choice of thermodynamic constants and metabolite concentration bounds), so our choice is mainly justified by the need to pick a single reference solution for the MDF analysis.

5) Escher maps are already available for e.coli metabolism. What is the key contribution of the new maps build here?

At the time of writing, the metabolic maps publicly available in the Escher web app (https://escher.github.io/) cover either core/central metabolism or individual subsystems, such as the metabolism of nucleotides and saturated fatty acids. The Escher metabolic map we provide with *i*CH360 covers simultaneously core and biosynthetic metabolism, displaying how different biomass building blocks (amino acids, nucleotides, and fatty acids) are synthesised from core precursors. They will also be valuable resources for educational use in systems biology. Additionally, the compactness of our model allows it to be visualised in full without sacrificing interpretability, allowing for the inspection of simulation results or mapped experimental data in a single, cohesive map.

Minor comments:

1) There are lot of spelling mistakes in the paper. Few examples: Line 172: estensive; Line 189: catalyses; Supplemental section A3: man text.

We thank the reviewer for pointing them out. These have been fixed in the revised manuscript.

2) Line: 226. Which section are the authors referring to?

We apologise for the presence of broken reference(s) in the text. All references to individual subsections have been removed in the revised version of the manuscript following the journal requirements.

3) The authors use COBRApy not COBRA toolbox. Modify it in lines 82 and 459.

This has been fixed in the revised text.

4) Italics was used very often in unnecessary places

We have removed the occurrences of italics used for emphatic purposes, and maintained it only for gene / species names and Latin expressions (*de novo*, *ad hoc*, etc.).

5) Most of the core (or compact) models developed are for prokaryotic models especially e.coli models. Can the authors discuss how these methods can be extended to apply on eukaryotic models?

While various pipelines we generated to annotate, parametrise, and validate the model (which we share in our supporting code repository) may be reused in other scenarios, this work does not intend to propose a general methodology for the generation of core/compact metabolic models. Instead, we aimed to focus on the final model and showcase its properties, annotations, and applications to interested users. In our view, the challenges of extending this approach to more complex (e.g. eukaryotic) metabolic models would be highly case-specific and dependent on the existing annotation and literature on the organism. In this light, we believe that building a systematic pipeline for the generation of meaningful core models would likely benefit from the synergistic use of algorithmic reduction tools (such as https://doi.org/10.1186/s12918-015-0191-x), expert domain knowledge, and iterative rounds of both automated and manual curation/annotation of the model.

Reviewer #2:

This is an elegant and well-written paper that presents an expertly curated, medium-scale metabolic model of Escherichia coli. The quality of the work is impressive, and the resulting model will undoubtedly support numerous future applications. My comments have been uploaded as an attachment.

We thank the reviewer for the positive evaluation on the manuscript.

Major-importance comments

• The catalytic disruption analysis and the associated interpretations need to be clarified (see the associated comments below).

See our responses below.

Medium-importance comments

• Lines 161-171: You don't detail the KEGG annotation. It would be interesting for the reader to know a bit more about it here (as you display some information about it on figure 3A)

This information has been added in the revised text (L157-158).

• Lines 203-219 and Method's section lines 505-518: the catalytic disruption analysis is not clear to me. If I understand correctly, you identify essential reactions for a given condition. Then, for each essential reaction, you individually knock-out the genes associated with it, and "propagate" the KO across the knowledge graph accordingly using the Boolean GPR rules you defined. You then "*catalogue the KO according to the reaction-level disruption it caused*" (and if the KO affects multiple essential reaction, you catalogue it using the strongest disruption). Finally, you assign a fitness for each KO-disruption categorycondition tuple. If I am right and this is what you do, then I believe your method section is not super clear. I would suggest the following clarifications, or something similar:

Suggested clarification for results section: "*For each growth condition, we identified essential reactions by simulating in silico knockouts of reactions and selecting those that abolish growth. For each such reaction, we identified all catalyzing genes via our knowledge graph. We then performed simulated knockouts for each gene, propagated the disruption through the graph using Boolean GPR logic, and assessed the impact on the reaction(s). Based on which catalytic edges were lost, we assigned a disruption category to the KO as follows: (i) complete (all catalytic edges lost), (ii) full primary (only primary edges lost), (iii) partial primary (some primary edges lost), or (iv) secondary (only secondary edges lost). If a single gene knockout disrupted multiple reactions, we assigned the most severe disruption among them. These gene-condition-disruption tuples were then mapped to corresponding experimental fitness values from the Price et al. (2018)*."

Suggested clarification for methods section: "*To simulate catalytic disruptions, we first identified condition-specific essential reactions using growth simulations. For each essential reaction, we enumerated associated genes and simulated their individual knockouts. Knockouts were propagated across the knowledge graph using Boolean logic rules, deactivating proteins and reactions as appropriate. Each resulting gene-condition pair was labeled with a disruption class depending on the type of enzyme-reaction associations lost: complete, full primary, partial primary, or secondary. If a gene knockout affected multiple reactions, we labeled the gene with the most severe disruption across all affected reactions. Finally, we compared predicted disruption types to measured fitness values from*

Price et al. (2018), using Wilcoxon rank-sum tests to assess statistical differences in fitness between disruption classes."

Indeed, the reviewer's understanding of our procedure is correct, and we thank them for the suggestions on how to clarify this section. We have implemented the suggested changes (with some minor differences) in the revised manuscript, see L201-213 (Results) and L578-600 (Materials and Methods).

Even if I get the analysis correctly, a strong limitation is that you do not simulate the weaker effect of secondary catalysis by using e.g. lower values for reaction boundaries in the GEM model. This should be discussed and balance your statement lines 218-219.

With our catalytic disruption analysis, we did not intend to provide a "forward simulation" type of analysis, in which the model is used to simulate a given phenotype (fitness) in response to a perturbation (a gene knockout). This is because the catalytic annotation we curated is of qualitative nature and does not provide information about what fraction of an enzymatic activity is performed by different isozymes. Instead, our analysis allowed us to map this qualitative information (via the assignment of a disruption category to each knockout) to experimentally measured quantitative data. Of course, an *ad-hoc* heuristic could be designed for such a forward simulation (e.g. tightening flux bounds by predefined factors depending on the nature of the catalytic disruption caused by the KO), but it is unclear what the quantitative value of this would be given the arbitrary choice of scaling factors for the flux bounds.

In this light, we now discuss this aspect in the revised text (L223-226).

• L304-328 (Saturation FBA): Why did you only perform satFBA for glucose? Could you do it/would it be interesting to do it for other substrates (modulo the calibration of the Km values)?

We presented the satFBA analysis as a possible example application of our model. In this light, we believe that replicating the analysis for other substrates would be outside of the scope of the manuscript. However, the code to run the satFBA analysis is freely available in the repository supporting the manuscript (see https://github.com/marco-corrao/iCH360_paper/tree/main/Analysis/satFBA), so the interested reader can easily reproduce the results for any growth condition of choice.

• Line 507: "*a small number of known false positives*": please provide the list as supplementary

The two reactions in question – and the reason for their essentiality in our model, but not in practice – are now explicitly mentioned in the revised text at L583-589 (see also our response to the similar comment by Reviewer 1).

• Table 3: Please define Kmax_app and Kapp, and add their units

We have now amended the table to clarify that our turnover numbers are in units of s^{-1}.

• Would it make sense to obtain a MEMOTE score of the model?

We have run MEMOTE on our model, and use the output to correct for few missing gene annotations and SBO terms. We obtained a final score of 89% and have included the MEMOTE report in the repository supporting the manuscript:

https://github.com/marco-corrao/iCH360_paper/blob/main/Model/iCH360/iCH360_memote_report.html

Minor-importance comments

• L13-14: "*We enriched the stoichiometric network with extensive biological information and quantitative data, enhancing the scope and applicability of the model*"→ You could add few examples of these biological information and quantitative data for the reader in the abstract here: "[…] *quantitative data (e.g., such as thermodynamics, kinetic constants, etc.)*"

This information has been added in the abstract of the revised text.

• L38: And how many metabolites? Please add this information

1877 metabolites. The information has been added in the revised text (L14).

• Fig 1: "*The map was created the metabolic*"→ "with" is missing

This has been fixed.

• Line 109-110: please provide an estimated value (e.g. average percentage difference over all conditions) of the differences between the models. Why don't you test/show all the carbon sources that the models have in common?

In the revised text, we have extended Supplementary Figure S6 to now include growth on malate, gluconate and glycerate. Across all carbon sources and conditions tested, we observed an average

relative percentage difference of 2.3%, computed using a cut-off of 0.05 h^{-1} (for a carbon source uptake flux of 10 mmol/GDW/h), below which a model was considered unable to grow. This figure is reported in in L105-107.

• Line 121: “yield”: please define what yield you refer to here (as different definitions of yield can be used, and it can be confusing for the reader). Also, please specify how the reader can infer the yield from the production envelope (for better clarity).

Here we referred to yield in terms of moles of product per mole of carbon source. This clarification has been in the revised text (L120). Since the production envelopes were generated using a fixed uptake rate for the carbon source of 10 mmol/gDW/h, the yield can be inferred by dividing the production flux (y-axis of the envelope plots) by 10. This information has been added in the caption of Fig. 2 for clarity.

• Line 137 and Supplementary figure S7: “B” is missing

This has been fixed.

• Line 172: “estensive”→ please correct

This has been fixed.

• Line 226: “(*Section)”* → please correct

We apologise for the presence of broken reference(s) in the text. All references to individual subsections have been removed in the revised version of the manuscript following journal requirements.

• Figure S16 and S17: “*(Section)”* → please correct. Also, you could use a more contrasted color scale for better clarity

Since the journal template does not include subsection numbering, we have now removed cross-references to specific subsections in the revised text. Further, we have updated Supplementary Figures S16 and S17, where we now use a nonlinear color map to improve clarity.

Reviewer #3:

Recommendation: Accept with Minor Revisions

General Comments:

This manuscript presents a nicely developed, annotated stoichiometric model for E. coli, enriched with well-motivated additions such as thermodynamics and resource allocation modeling. It represents a valuable contribution to the metabolic engineering community and broadens the toolkit available for metabolic modeling studies.

We thank the reviewer for the support and helpful comments on the manuscript.

Specific Comments and Suggestions:

Abstract:

- L9: The text mentions E. coli K-12 MG1655. Since the model is reduced, its applicability likely extends beyond this strain. Consider emphasizing the broader applicability and potential for adaptation to other model organisms with suitable modifications.

We have mentioned the broader applicability of the model beyond the K12 MG1655 strain in our Discussion (L438-440).

- Query: Address if the model simulations replicate the growth defects observed in the study by [Reference: 10.1016/j.cels.2020.10.011] with CRISPRi targeting CysH, MetE, Ppc, and Pts.

We have run this analysis by considering a reference pFBA distribution computed on the WT model and simulated each knock-down, heuristically, by bounding the flux of the affected reactions (PAPSR, METS, PPC, GLCptspp, corresponding to target genes *cysH*, *metH*, *ppc*, and *pts*, respectively) by a fifth of the flux in the WT reference distribution. This tightening of the flux bound was chosen in line with the average 5-fold reduction in target enzyme abundance reported in the study as a result of CRISPRi knockdown. Our results qualitatively recapitulate observed growth defects for 3 of the four reactions (PAPSR, METS, GLCptspp), for which the simulation predicted a reduction in maximal growth rate of ~80%. We stress, however, that buffering mechanisms described in the article to explain measured growth defects involve metabolite pool rearrangement and transcriptional regulation effects, which simply cannot be captured by a stoichiometric model like ours. Hence, a quantitative comparison with this experimental data is not necessarily possible.

On the other hand, the simulated knockdown of PPC resulted in negligible growth defects, opposite to experimental observations in the study. In this regard, we note that *ppc*, experimentally known to be an essential gene for growth on glucose, is a false-positive (its knockout does not prevent the model from

predicting growth) in various *E. coli* metabolic models, including ours (*i*CH360) and its genome-scale parent (*i*ML1515). This mismatch between experimental model predictions and experimental observations with regard to *ppc* has been noted and investigated before (https://doi.org/10.1128/mbio.02259-20). Briefly, this behaviour results from the fact that the models predict activation of the glyoxylate shunt to circumvent the deletion and replenish the oxaloacetate pool, but this does not seem to be viable *in vivo*, likely due to regulatory effects that are not accounted for by these models.

- L18: While comparisons to genome-scale models are mentioned, what are the advantages over the core (ECC/ECC2) model.

The difference with ECC is mainly a matter of metabolic scope: *i*CH360 includes the biosynthetic pathways required to produce the main biomass building blocks, which are not part of ECC (see L37-39 in the revised text). The difference with ECC2 is both methodological (our model was built manually and bottom up, rather than algorithmically reducing a larger model) and structural (the metabolic space of ECC2 reaches complex biomass components, while *i*CH360 only reaches biomass building blocks). These differences are discussed in greater detail in L88-94.

Minor comments:

- L50: Substantiate the claim regarding "difficulties with constraints in genome-scale" models by citing relevant literature, such as:

- [Reference: https://doi.org/10.1016/j.copbio.2015.08.021]

- [Reference: https://doi.org/10.1016/j.mib.2010.03.001]

- Include references for refined genome-scale models:

- [Reference: 10.1529/biophysj.106.093138]

- [References: https://doi.org/10.1007/978-1-4939-1170-7_3], etc.

We now added a citation to Henry et al. (2007) to the text (L27):

"More complex methods, including sampling of metabolic flux distributions [12], elementary flux mode (EFM) analysis [13], thermodynamics-based metabolic flux analysis [14], or kinetic modeling can be used to gain additional insight into the governing principles and constraints of microbial metabolism, but are difficult to apply to large models."

- L71: Clarify the term "effective" in the context of biomass-producing reactions; consider replacing it with "compact" or "lumped" for precision.

We have replaced the term “effective” with “compact” in the revised manuscript (L58).

- L87: Explain why not all five nucleotides were included in the model.

This was a typo, and our model indeed includes all five nucleotides. We thank the reviewer for spotting this and have corrected it in the revised text (L76).

- L126-137 and L141: Consider moving detailed analysis and figures to supplementary materials to maintain the flow of the main text. Confirm if thermodynamic statements were verified by calculations.

We have moved the details of the investigation of acetate production discrepancies between the models into a dedicated supplementary section (Section 3 and Figure S7 in S1 Text). We verified our claim that the reverse operation of POR5 reaction (in the direction of pyruvate production) is thermodynamically unrealistic under ambient conditions based on component contribution estimates of the reaction’s thermodynamics, obtained via eQuilibrator (https://equilibrator.weizmann.ac.il/). Even assuming a redox potential of *E. coli* flavodoxin of -500 mV, it would still not be enough to drive the reaction in the direction of CO_2 fixation at standard conditions ($\Delta G'0 = +3.9$ kJ/mol). In ambient $CO_{2,}$ the $\Delta G'$ would even be higher.

- Fig. 2: Comment on the high growth rate of 0.9 in minimal medium. Consider expressing the x-axis in yield terms for better comparison with experimental data.

The production envelope analysis was performed, as per convention, by fixing the upper bound on carbon source uptake flux to a predefined value (here, 10 mmol/gDW/h, a standard “default” value in the metabolic modelling community), Hence, the maximal growth rate of 0.9 h^{-1} results from the (arbitrary) choice of this bound. In the absence of fixed maintenance constraints, the predicted biomass yield (in gDW/mmol carbon source) and production yield (in mmol product/mmol carbon source) would be independent of the choice of uptake flux, and could conveniently be plotted instead of growth rate/production flux to generate the production envelope. However, the presence of fixed maintenance costs (the lower bound on the ATP hydrolysis reaction, ATPM, inherited from the gnome-scale parent), makes these yields dependent on uptake flux, so that the production envelope, even if expressed in terms of yield, would still be contingent on the choice of uptake flux. We have now extended the caption of Figure 2 to clarify how the biomass and production yields can be deduced from the plot. However, considering these plots are meant to highlight relative differences between the models, rather than

absolute values, we chose to maintain the plot in terms of growth rate, which we believe to be more intuitive to most readers.

- Fig. 3D: Define the black dots and clarify fitness definition. Was it growth rate in relation to WT growth?

The black dots are outliers in the box and whiskers plot and are conventionally defined as points located above (below) 1.5 times the third (first) quartile of the data. We have now explicitly stated this in the figure caption.

The fitness measurements from Price et al (2018) are based on competitive fitness assays and, briefly, reflect the normalised logarithmic ratio of mutant counts (as assayed from molecular barcode counts) between the start and end of a growth experiment (see the original study for more details: https://doi.org/10.1038/s41586-018-0124-0). Since the precise definition of these fitness values is not relevant to our conclusions, we believe that reporting these experimental details in our manuscript would be superfluous.

- "Primary and secondary catalysis": Specify conditions, and clarify if there was a quantitative cut-off.

We have now explicitly stated the growth conditions over which our validation with experimental data was performed (L583). Our primary/secondary catalysis annotation is qualitative in scope, and there was no strict quantitative threshold used to annotate catalytic relationships as secondary. Ultimately, the validation of our annotation against a large independent dataset of fitness measurements is what justifies its validity.

- L230: Explain the treatment of lumped reactions concerning turnover numbers in both the model and experimentally.

With the exception of the biomass-producing reaction, which is not associated to any enzyme constraint, our network does not include lumped pathways. In the enzyme-constrained model, every enzymatic reaction is simply associated with one catalysing enzyme (see Methods, *Construction of the enzyme-constrained metabolic model* for more details). Lumped pseudo-reactions mentioned in L535 are just used for visualisation in the compressed Escher map, but the model itself contains all reactions. Similarly, when substrate channeling is mentioned in the context of thermodynamic analysis (L407), we suggest lumping of reactions as a way of resolving thermodynamic anomalies (should it be needed for a specific analysis), but did not implement these changes in the model.

- L241: Describe the "custom heuristic" method or approach.

Our parameter fitting procedure is explained in great detail in Section 6 of S1 Text and outlined in the Methods (see Adjustment of turnover numbers across conditions). We believe that further diving into the details of the procedure in the Results section would hamper readability and shadow the key outcomes of this part of the work.

- Fig. 4: Elaborate on how lumped pathways are represented and discuss observed shifts in enzyme abundance predictions vis-à-vis experimental data.

With the exception of the biomass-producing reaction (which is not involved in any enzyme constraint), our model does not include any lumped reaction, so each data point in the figure represents a prediction/measurement tuple for a given enzyme.

- Fig. 5 & 6: Address discrepancies in growth rates observed across figures, clarify assumptions on maintenance, and suggest consistent units (e.g., g/gGlc) for ease of comparison.

Assuming that the reviewer is referring to discrepancies in maximal growth rates between the production envelope analysis (Fig. 2) and the analyses based on enzyme constraints (Fig. 5,6), these are simply the results of two alternative modelling approaches. The production envelopes are based on stoichiometric modelling (FBA), where the uptake flux is bounded by design to a desired value, and the predicted maximal growth rate is effectively limited by the choice of this bound. In enzyme-constrained modelling, the substrate uptake rate is left unbounded, and the computed maximal growth rate is effectively limited by the enzyme pool constraint imposed on the model. To avoid misunderstanding, we have clearly stated what the underlying assumptions are in each modelling scenario (fixed uptake flux in the former, fixed enzyme budget constraint in the latter) below the relevant figures.

Regarding the second point, we have now updated Fig. 6 so that both figures use the same units for yield (gDW/mol glucose). We thank the reviewer for noticing this discrepancy.

- L363: Clarify how substrate channeling influences thermodynamics. Discuss potential reaction coupling mechanisms.

In the revised text, we have clarified the meaning of substrate channelling and how it effectively relates to model thermodynamics (L404-409). Potential coupling mechanisms for reactions in the model have been discussed in detail in https://doi.org/10.1093/bioinformatics/btab194, and we now explicitly refer the reader to this work for more details on individual reactions.

- Table 1: Explain the exclusion of arabinose, despite its significance in synthetic biology.

While arabinose is frequently used in synthetic biology as a chemical inducer for synthetic gene circuits, its use as a carbon source is, to our knowledge, less relevant. As this is a stoichiometric model, the simulation of effects such as inducible transcription is outside the scope of the model.

Technical:

- Ensure the abbreviation list is comprehensive, particularly for enzymes.

As the Plos template does not include a list of abbreviations, the list has been removed from the revised manuscript.
